# ATP and MO25α Regulate the Conformational State of the STRADα Pseudokinase and Activation of the LKB1 Tumour Suppressor

**DOI:** 10.1371/journal.pbio.1000126

**Published:** 2009-06-09

**Authors:** Elton Zeqiraj, Beatrice Maria Filippi, Simon Goldie, Iva Navratilova, Jérôme Boudeau, Maria Deak, Dario R. Alessi, Daan M. F. van Aalten

**Affiliations:** 1 Division of Molecular Microbiology, College of Life Sciences, University of Dundee, Dundee, Scotland; 2 MRC Protein Phosphorylation Unit, College of Life Sciences, University of Dundee, Dundee, Scotland; 3 Division of Biological Chemistry and Drug Discovery, College of Life Sciences, University of Dundee, Dundee, Scotland; Stanford University, United States of America

## Abstract

Pseudokinases lack essential residues for kinase activity, yet are emerging as important regulators of signal transduction networks. The pseudokinase STRAD activates the LKB1 tumour suppressor by forming a heterotrimeric complex with LKB1 and the scaffolding protein MO25. Here, we describe the structure of STRADα in complex with MO25α. The structure reveals an intricate web of interactions between STRADα and MO25α involving the αC-helix of STRADα, reminiscent of the mechanism by which CDK2 interacts with cyclin A. Surprisingly, STRADα binds ATP and displays a closed conformation and an ordered activation loop, typical of active protein kinases. Inactivity is accounted for by nonconservative substitution of almost all essential catalytic residues. We demonstrate that binding of ATP enhances the affinity of STRADα for MO25α, and conversely, binding of MO25α promotes interaction of STRADα with ATP. Mutagenesis studies reveal that association of STRADα with either ATP or MO25α is essential for LKB1 activation. We conclude that ATP and MO25α cooperate to maintain STRADα in an “active” closed conformation required for LKB1 activation. It has recently been demonstrated that a mutation in human STRADα that truncates a C-terminal region of the pseudokinase domain leads to the polyhydramnios, megalencephaly, symptomatic epilepsy (PMSE) syndrome. We demonstrate this mutation destabilizes STRADα and prevents association with LKB1. In summary, our findings describe one of the first structures of a genuinely inactive pseudokinase. The ability of STRADα to activate LKB1 is dependent on a closed “active” conformation, aided by ATP and MO25α binding. Thus, the function of STRADα is mediated through an active kinase conformation rather than kinase activity. It is possible that other pseudokinases exert their function through nucleotide binding and active conformations.

## Abstract 2

The conformation of the pseudokinase STRADα, which is regulated by binding to ATP and to the scaffolding protein MO25α, is key to the activiation of the LKB1 tumor suppressor complex.

## Author Summary

There are 518 human protein kinases that are responsible for orchestrating the phosphorylation-dependant signal transduction events that regulate almost all cellular processes. Curiously, approximately 10% of protein kinases lack one or more catalytic residues, and these kinases have been termed pseudokinases. It has been proposed that some pseudokinases act as scaffolds, bringing together proteins involved in signalling networks. Here, we report the structure of the pseudokinase STRADα in complex with the adaptor protein MO25α; together these two proteins regulate the LKB1 tumour suppressor kinase. Despite lacking several key catalytic residues, STRADα binds ATP and adopts an active conformation typical of catalytically competent kinases. The affinity of STRADα for ATP is enhanced by MO25α and vice versa. We go on to demonstrate through mutagenesis studies that binding to both ATP and MO25α is essential for the activation of LKB1. Our data suggest that STRADα exerts its functions through an active conformation, not through actual catalytic activity, thus raising the possibility that pseudokinases regulate signalling networks by adopting different structural conformations.

## Introduction

Pseudokinases are classified as protein kinases that lack key catalytic residues within their kinase domain [1],[2]. These proteins are emerging as important regulators and scaffolding components of various signal transduction networks [2]. Despite being predicted to lack intrinsic kinase activity, several “pseudokinases” such as WNK, CASK, and IRAK2 still possess the ability to phosphorylate substrates. In the case of WNK isoforms, the missing conserved catalytic Lys residue in subdomain-II is substituted by another Lys residue located in subdomain-I [3]. CASK, despite lacking the conserved Mg^2+^ binding Asp residue in the DFG motif of subdomain-VII, folds into an active conformation capable of binding ATP and phosphorylating substrates in the absence of Mg^2+^ ions [4]. Interestingly, recent studies have shown that mutations in CASK affect brain development and cause mental retardation in humans [5]. Recent data also indicate that the IRAK2 pseudokinase, despite lacking the Mg^2+^ binding DFG motif as well as the catalytic HRD motif, still possesses activity [6]. These results suggest that some of the other proteins in the human genome that are classified as pseudokinases may still possess catalytic activity and thus function as normal kinases.

The STe-20 Related Adaptor (STRAD) pseudokinase forms a 1∶1∶1 heterotrimeric complex with the LKB1 tumour suppressor kinase and the scaffolding protein MO25 [7],[8]. In humans, there are two closely related isoforms of STRAD (STRADα and STRADβ) and MO25 (MO25α and MO25β) that similarly interact with and activate LKB1. Loss-of-function mutations in the LKB1 kinase in humans result in the inherited Peutz-Jeghers cancer syndrome [9]. Inactivating mutations in LKB1 are also increasingly being reported in sporadic cancers, in particular lung cancer [10]. LKB1 exerts its tumour-suppressing effects by phosphorylating and activating AMP-activated protein kinase (AMPK) as well as a number of other related kinases [11]. LKB1-mediated activation of AMPK occurs when cellular energy levels are low, and activation of AMPK inhibits cell growth and proliferation through multiple pathways, including suppressing activity of mTOR [12],[13].

Recently, it was reported that a severe human developmental and epileptic syndrome termed polyhydramnios, megalencephaly, symptomatic epilepsy (PMSE), was caused by a homozygous partial deletion in the STRADα gene (*LYK5*), truncating 180 C-terminal residues of the protein [14]. Individuals affected by this condition suffer from severe mental retardation, gross movement disorders, and childhood mortality [14]. How this mutation affects STRADα function and its ability to interact with LKB1 is unknown, although histological staining of neuronal tissues of PMSE patients has suggested elevated mTOR pathway activity, which could potentially result from loss of LKB1 kinase activity.

Unlike the majority of kinases that require phosphorylation of their T-loop, LKB1 is activated through direct interaction with STRADα/β isoforms [7],[8]. The kinase domain of LKB1 binds to the pseudokinase domain of STRAD [7]. At least 12 point mutations located in the LKB1 kinase domain that prevent LKB1 from interacting with STRAD isoforms have been identified in human cancers [15]. Activation of LKB1 and interaction with STRAD isoforms is markedly enhanced in the presence of MO25α/β isoforms, indicating that MO25 stabilizes the interaction between STRAD and LKB1. The C-terminal Trp-Glu-Phe residues (WEF motif) of STRADα bind to MO25α, and mutations of these residues abolish this interaction [8]. Structural analysis of MO25α revealed a helical repeat, horseshoe-shaped protein that interacts with the WEF motif of STRADα through a hydrophobic pocket located on its convex C-terminal surface [16]. In contrast, proteins that are distantly structurally similar to MO25α, such as the Armadillo repeat proteins PUM1, β-catenin, and importin-α, interact with their binding partners through their concave surface [17]–[19]. Many of the surface-exposed residues on the MO25α concave surface are conserved between species, suggesting that these may mediate interactions with (an) unknown regulator(s) [16]. Although STRADα mutants lacking the C-terminal WEF motif are unable to interact with MO25α alone, they can still form a heterotrimeric complex with LKB1 and MO25α, demonstrating that STRADα possesses additional interactions with LKB1 and/or MO25α, separate from the WEF motif [15].

All studies undertaken to date suggest that STRADα expressed in bacteria is incapable of autophosphorylating or phosphorylating other substrates tested (MBP, histone 2A, or LKB1) when assays were undertaken in the presence of Mg^2+^ ions [7],[15] (J. Boudeau, unpublished data). Despite lacking detectable kinase activity, STRADα is still capable of interacting with ATP as well as ADP in a magnesium-independent manner [15]. Mutations that abolish ATP binding do not affect the ability of STRADα to activate LKB1 in the presence of MO25α. Thus, the role of ATP-binding to STRAD is unclear.

Here, we report the structure of STRADα as part of the STRADα/MO25α heterodimer. The data show that despite being inactive, STRADα folds into an ATP-bound, closed conformation with an ordered activation loop similar to that of fully active protein kinases. Our data establish that STRADα is indeed deficient in intrinsic catalytic activity because it lacks most essential catalytic residues. Moreover, we observe that STRADα does not only interact with MO25α through its WEF motif as previously envisaged, but forms an extensive network of interactions with the highly conserved concave surface of MO25α. Binding studies and mutagenesis data show that the closed/“active” conformation that STRADα assumes is maintained through cooperative binding of ATP and MO25α. STRADα mutants incapable of interacting with ATP and MO25α are unable to activate LKB1, despite interacting with it. We conclude that the ability of STRADα to activate LKB1 is dependent on an active conformation rather than catalytic phosphoryltransferase activity. Our results also indicate that the human mutation that causes PMSE syndrome destabilizes STRADα and prevents it from binding to, and activating LKB1.

## Results and Discussion

### STRADα Adopts the Canonical Kinase Fold

**Figure 1:**
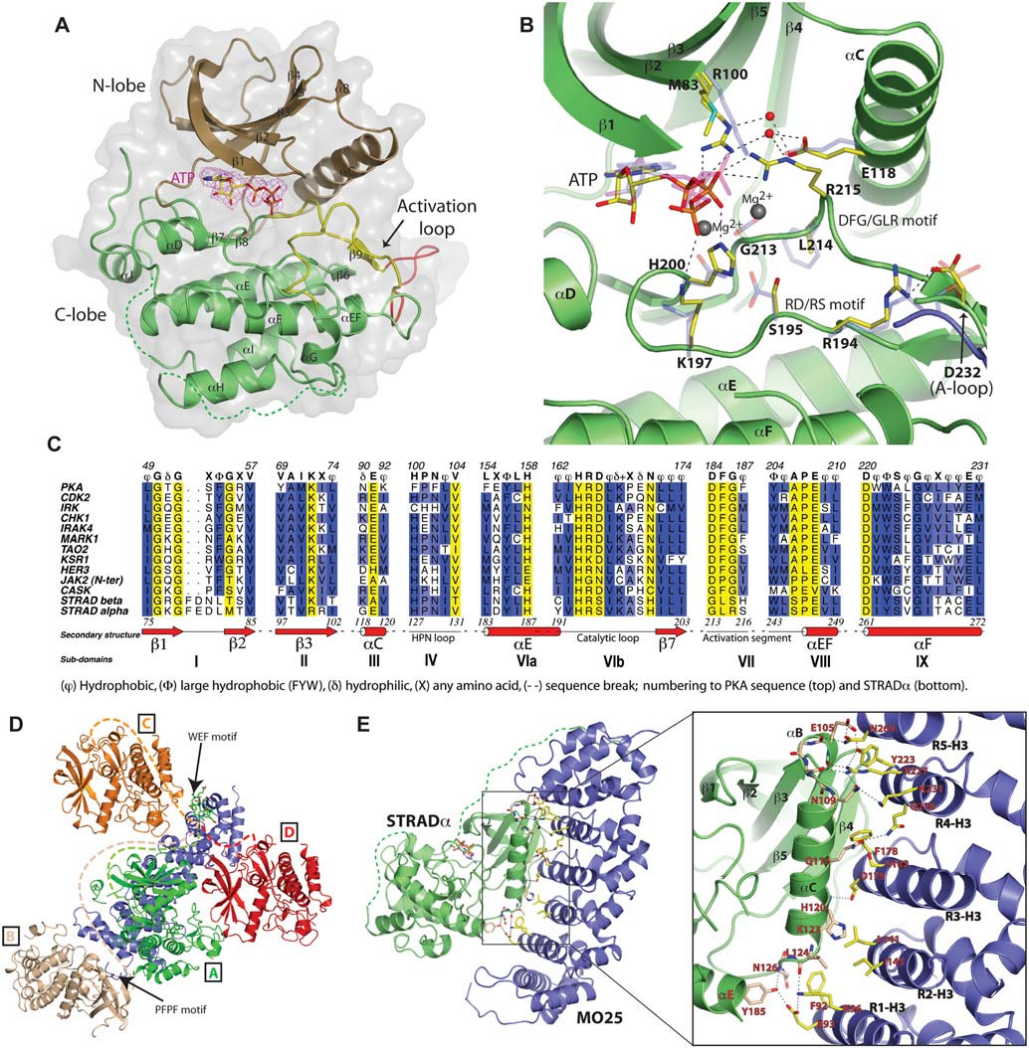
STRADα structure, active site, sequence motifs, and interactions with MO25α. (A) Overall structure of STRADα shown in cartoon representation (N-terminal lobe coloured brown, C-terminal lobe coloured green) with transparent molecular surface. For clarity, the WEF motif has been omitted. Secondary structure elements are labelled according to the structure of PKA [22]. The activation loop is coloured yellow, with the section that appears to be unique to STRADα/β (residues 221–229) coloured red. The ATP molecule is shown in stick representation, and an unbiased *F*_o_-*F*_c_ electron density map is shown in magenta, contoured at 2.5σ. Dotted lines represent regions that were not well defined by electron density and are not included in the refined model. (B) Superposition of the STRADα and TAO2 (PDB ID 1U5R [23]) active sites, highlighting key residues required for activity. STRADα residues (labelled) are shown as stick models with yellow carbon atoms; the corresponding TAO2 residues are shown with blue, transparent carbon atoms. Water molecules are represented by red spheres, and gray spheres represent Mg^2+^ ions from the TAO2 structure. The glycine-rich loop and part of the activation loop have been omitted for clarity. (C) Multiple sequence alignment of STRADα and other pseudokinases, highlighting (in yellow) key motifs that are normally essential in active eukaryotic protein kinases. (D) Crystallographic contacts between MO25α (blue) and symmetry-related STRADα molecules shown in cartoon representations. The STRADα WEF motif bound to MO25α is shown as sticks with green carbons. Dashed lines represent the distance from the last residue of the C-terminal lobe of each STRADα molecule able to donate the WEF motif, corresponding to 52, 83, 65, and 55 Å (straight-line distances) for molecules A, B, C, and D, respectively). An additional tight crystallographic contact, through the MO25α N-terminus (“PFPF motif”) is also indicated and further discussed in Figure S3B–S3F. (E) Structure of the STRADα (green) MO25α (blue) complex. Residues that make direct contact are shown as sticks, with hydrogen bonds shown as dotted black lines.

STRADα comprises a pseudokinase domain (residues 58–401), two nuclear export sequences (residues 21–29 and 417–426) [20], and a C-terminal WEF motif (residues 429–431) previously shown to interact with MO25α [8],[16]. We focused on the interaction between the STRADα pseudokinase domain (residues 59–431) and full-length MO25α (residues 1–341). These proteins were coexpressed in *Escherichia coli* and the STRADα/MO25α complex eluted as a heterodimer of the expected size from a gel filtration column, yielding approximately 60 mg of the complex from 4 l of culture (Figure S1). Initial crystals of the STRADα/MO25α complex in space group P2_1_2_1_2_1_ diffracted only to 4.8 Å resolution (Table 1). With the help of chemical lysine methylation [21], diffraction of these crystals (retaining the same space group and unit cell dimensions) improved to 2.35 Å (Table 1). The structures of both methylated and unmethylated crystals were solved by molecular replacement and revealed the same packing/intermolecular interactions. The high-resolution, methylated form of the complex was refined to a final model with good statistics (*R*_free_/*R*_work_ of 0.254/0.206; Table 1). The structure of STRADα exhibits the classical bilobal protein kinase fold, with the N-terminal lobe (residues 59–152) organized around a central β-sheet, and a C-terminal lobe (residues 153–401) that is largely α-helical (Figure 1A). A well-resolved molecule of ATP was observed in the cleft between the small and large lobes of the pseudokinase (Figure 1A and 1B). The ATP molecule displays the canonical binding mode and retains a similar conformation to that of ATP molecules bound to active kinases (root mean square deviation [RMSD] = 0.9 Å on all atoms compared to ATP bound to PKA [22]).

**Table 1:** Summary of data collection, structure refinement, and analysis.

Parameter	Subparameter	Native	Methylated
**Space group**		P2_1_2_1_2_1_	P2_1_2_1_2_1_
**Unit cell (Å)**	*a* = 73.3	—	73.7
	*b* = 83.5	—	82.9
	*c* = 134.3	—	134.3
**Molecules/asu**	STRADα	1	1
	MO25α	1	1
**Resolution (Å)**		20–4.8 (4.97–4.80)	20–2.35 (2.48–2.35)
**Observed reflections**		13,019	190,928
**Unique reflections**		4,229 (409)	34,668 (4,988)
**Redundancy**		3.0 (3.0)	5.5 (5.6)
***I*/σ*I***		13.6 (1.9)	13.4 (2.9)
**Completeness (%)**		98.9 (97.1)	99.6 (99.9)
***R*_merge_**		0.094 (0.487)	0.100 (0.617)
***R*_work_, *R*_free_**		—	0.206, 0.254
**RMSD from ideal geometry**	Bonds (Å)	—	0.011
	Angles (°)	—	1.277
***B*** **-factor RMSD (Å^2^)**	(Backbone bonds)	—	1.063
**Average ** ***B*** **-factor (Å^2^)**	Protein	—	28.59
	Ligand (ATP)	—	34.46
	Water	—	28.36
**Ramachandran plot statistics (%)**	Most favoured region	—	92.4
	Additional allowed region	—	6.7
	Generously allowed region	—	0.7
	Disallowed region	—	0.2

Values for the highest resolution shell are given in parentheses.

asu, asymmetric unit.

### STRADα Binds ATP Using a Mg^2+^-Independent Mechanism

Sequence comparison reveals that STRADα lacks numerous essential catalytic residues found in active protein kinases, namely a conserved Gly residue in the glycine-rich loop (subdomain-I), the Lys residue of the VAIK motif (subdomain-II), the catalytic Asp residue of the HRD motif (subdomain-VIb), a conserved Asn residue (subdomain-VIb), as well as the entire DFG motif in subdomain-VII (Figure 1B and 1C). Despite missing these key residues, STRADα adopts a similar overall conformation to that of TAO2 (sharing 25% sequence identity and 37% sequence similarity), an active protein kinase of known structure [23] (RMSD = 1.4 Å on 197 Cα atoms). Comparison of the STRADα and TAO2 structures reveals that a number of substitutions of key catalytic residues are found in STRADα. Met83 replaces one of the conserved Gly residues in the glycine-rich loop, Arg100 substitutes the catalytic Lys residue in the VAIK motif, Ser195 replaces the Asp residue in the HRD motif, His200 substitutes for the conserved Asn in subdomain-VIb, and the entire DFG motif is replaced by GLR (residues 213–215).

In active protein kinases, the DFG motif plays a pivotal role in coordinating two Mg^2+^ ions: one that orients the γ-phosphate into the position required for phosphoryl transfer and the other that controls ATP conformation by interacting with the β/γ phosphates. Consistent with the lack of the DFG motif in STRADα, no Mg^2+^ ions were observed in the STRADα-ATP complex, despite 1 mM MgCl_2_ being present in the crystallization mother liquor. However, despite the absence of Mg^2+^ ions, the positioning of the β/γ phosphates in STRADα was similar to that of active TAO2 kinase complexed to MgATP (Figure 1B). The β-phosphate is tethered through interactions with Arg215 from the GLR (DFG) motif, and His200 (subdomain-VIb), basic residues that may substitute for one of the positively charged Mg^2+^ ions (Figure 1B). The second Mg^2+^ ion and its coordinating residues are also missing; instead, the γ-phosphate only interacts with a conserved lysine (Lys197) in the catalytic loop. Thus, STRADα appears to have evolved a novel, Mg^2+^-independent mechanism to bind the phosphate groups of ATP. The presence of the two hydrogen bonds between N1 and N6 atoms of the ATP adenine ring and the protein backbone, observed in all active protein kinase structures, further illustrates the conservation of the ATP binding pocket. Thus, the STRADα structure explains previous observations that STRADα can bind ATP in the absence of Mg^2+^, and its similar affinity for ADP and ATP [15].

### STRADα Adopts an Active Conformation

Although the activation loop of STRADα (residues 212–245) is not phosphorylated, it is well ordered, a feature normally observed only in structures of activated protein kinases that are phosphorylated on their activation loop (Figure 1A). Remarkably, Asp232 in the activation loop occupies a position similar to the activating phosphorylated residue found in active kinases, e.g., (phospho)Ser181 in TAO2 (Figure 1B). Asp232 appears to play the same structural role as the activating phosphate group, coordinating the conserved arginine from the catalytic HRD motif (Arg194 in the STRADα HRS motif) (Figure 1B and 1C). Further evidence that STRADα adopts the canonical active conformation stems from the presence of a short antiparallel β-sheet between the β6 and β9 strands, which is a characteristic feature of the active state of kinases [24]. Furthermore, the STRADα αC-helix is rotated into the “closed” conformation found in active kinases [25],[26], with the conserved ion pair between the Glu118 on the αC-helix and Arg100 in subdomain-II formed via two water molecules (Figure 1B).

Despite STRADα binding ATP in the correct orientation for activity and folding into an active conformation, STRADα (residues 59–431) expressed in *E. coli* did not autophosphorylate or phosphorylate myelin basic protein (Figure S2). We have attempted to detect activity in the presence and absence of MO25α and/or 10 mM MgCl_2_. We have also generated mutations converting all the missing catalytic residues on the STRADα pseudokinase discussed above to the equivalent residues found in the active kinase TAO2 (Figure S2). However, none of these mutants showed autophosphorylation or phosphorylated myelin basic protein in the presence or absence of Mg^2+^ ions and/or MO25α (Figure S2). We also tested whether STRADα possessed ATPase activity, employing a highly sensitive ATPase assay kit (Innova Biosciences), but no activity was observed (E. Zeqiraj, unpublished data). Nevertheless, it is impossible to categorically rule out that STRADα will not, highly specifically, phosphorylate an as-yet unidentified substrate.

### Identification of the Biological STRADα/MO25α Complex

The asymmetric unit of the STRADα/MO25α complex crystals contains one molecule of MO25α, with a conformation similar to the previously published MO25α/WEF peptide complex structure [16] (RMSD = 0.6 Å on 292 Cα atoms), and one molecule of STRADα. The position and conformation of the WEF motif is similar to that in the previously described MO25α/WEF complex [16] (RMSD = 0.3 Å on 35 atoms, Figure S3A). Due to tight crystal contacts (total buried surface on MO25α by STRADα and its symmetry mates = 2,833 Å^2^), it was not immediately apparent which contacts represented biologically relevant interactions and which were crystallographic packing artefacts. Whereas clear electron density is present for the last six amino acids of STRADα (residues 426–431, including the WEF motif that interacts with MO25α, Figure S3A), residues 402–425 of STRADα were not visible in the electron density maps, and it was thus not possible to directly identify the appropriate symmetry mates of STRADα and MO25α that make up the biologically relevant binary complex. Analysis of the crystal contacts between symmetry-related molecules suggested that there were four possible ways in which STRADα could interact with MO25α (Figure 1D). We studied all four possible STRADα/MO25α complexes and ranked these in terms of total buried surface area, a possible method for distinguishing crystallographic from biological contacts [27]. Discounting the WEF motif interaction (800 Å^2^ buried surface area), identical in all four possible complexes, the buried surface area in each of the possible complexes is 1,550 Å^2^, 225 Å^2^, 58 Å^2^, and 200 Å^2^ for complexes A, B, C, and D, respectively (Figure 1D). In addition, the distances between the last well-defined residue of the STRADα C-terminal lobe and the first well-defined residue of the WEF motif at the extreme C-terminus of STRADα were measured for the four possible complexes. This yielded direct distances of 52, 83, 65, and 55 Å for complexes A, B, C, and D, respectively (Figure 1D). Taken together, it appears that complex A is the most likely biological interaction, since STRADα binds to the (highly conserved) concave surface of MO25α and has the largest buried surface area, while also possessing the shortest distance from the C-terminal lobe to the WEF motif (Figure 1D). Similarly, analysis of the possible complexes with PISA [28] yields the highest (1.0) complexation significance score (CSS) for complex A, while predicting that complexes B, C, and D will not be stable in solution.

The 6-His purification tag that extends from the N-terminus of STRADα (450 Å^2^ buried surface area in complex A) forms additional contacts between MO25α and STRADα. SPR studies demonstrate that His-tagged STRADα binds MO25α in vitro with the same affinity as STRADα lacking the His tag (Figure S4). Furthermore, MO25α residues 2–5 (Pro-Phe-Pro-Phe, termed the PFPF motif here) make hydrophobic contacts in a pocket adjacent to the STRADα ATP binding pocket on a symmetry-related copy of STRADα (Figure S3B). This is unlikely to constitute a physiological STRADα/MO25α interaction, as deleting this motif did not impair the in vivo interaction of MO25α with either STRADα alone or a complex of STRADα and LKB1 (Figure S3C and S3D). Moreover, we were unable to affinity purify overexpressed STRADα or LKB1 from a cell extract employing a PFPF motif containing biotinylated peptide (Figure S3E). A complex of LKB1/STRADα/MO25α(ΔPFPF) still activated the heterotrimeric AMPK complex expressed in *E. coli* with similar efficiency as wild-type LKB1/STRADα/MO25α (Figure S3F). Nevertheless, it is possible that the PFPF docking site on STRADα does play a role in enabling STRADα to interact with other regulators or substrates of the LKB1 complex. Intriguingly, a similar crystallographic interaction can be observed in the structure of the mammalian AMPK heterotrimeric complex [29]. In this case, a similar hydrophobic N-terminal motif “MYAF” from the β2 domain interacts with the kinase domain from the neighbouring molecule in the crystal lattice, albeit not near the phospho-nucleotide binding site.

### STRADα Interacts with the MO25α Concave Surface

**Figure 2:**
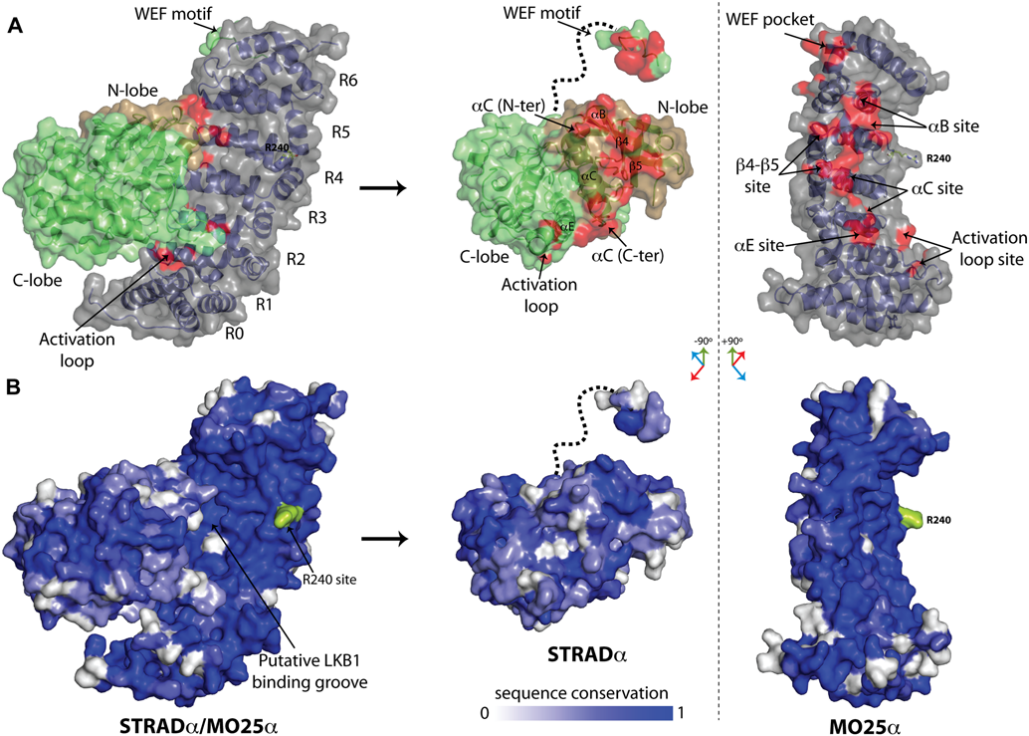
Sites of the STRADα/MO25α interaction and sequence conservation. (A) STRADα/MO25α complex and the interaction surface, as defined with the program CONTACT from the CCP4 package [42]. Surfaces of atom pairs closer than 3.9 Å are coloured red. The MO25α surface is coloured grey, and the N- and C-lobes of STRADα are coloured brown and lime green, respectively. Arg240 is shown as sticks. To aid visualization, on the right side of the figure, the complex is “opened up” by rotating the STRADα molecule about the vertical axes −90° and MO25α +90° with respect to the binary complex. (B) Sequence conservation (dark blue = conserved, white = not conserved) of STRADα and MO25α from *Caenorhabditis elegans* to *Homo sapiens* (sequence alignments provided in Figure S5). The putative LKB1 binding pocket and the Arg240 site are indicated with an arrow. STRADα and MO25α are shown in the same orientation as in (A) to aid visualization of conserved areas that are buried in the STRADα/MO25α complex.

MO25α is composed of seven structurally similar α-helical repeats (named R0–R6) that form a horseshoe-shaped molecule with a concave and a convex surface [16]. MO25α helical repeats R1–R6 consist of three α-helices (H1–H3) each, whereas repeat R0 consists of only two helices [16]. Helices H3 from repeat R1–R5 are arranged in an almost parallel fashion and make up the concave surface of MO25α (Figure 1E). Other helical repeat adaptor proteins, such as PUM1, β-catenin, and importin-α, make use of a similar concave surface to interact with macromolecular partners [17]–[19]. Strikingly, the crystal structure of the STRADα/MO25α complex reveals that, in addition to the interaction through the WEF motif, a major additional binding interface involves the STRADα N-terminal kinase lobe and the MO25α concave surface (Figures 1E and 2). Part of the interaction surface on STRADα is N-terminal to the αC-helix and comprises the loop between the αB/αC helices (residues 104–109), termed the “αB site” here (Figures 1E and 2A). This region forms an extensive hydrogen-bonding network centred on Arg227 from the R5-H3 of MO25α (Figure 1E), burying a total of 245 Å^2^ surface area. Residues Tyr223, Arg227, Lys231, and Asn269 of MO25α engage the side chains of residues Glu105 and Asn109 of STRADα, whereas Leu104, Ala106, Cys107, and Ser108 contribute to the interaction via their backbone atoms.

The αC-helix of STRADα runs along the concave surface of MO25α facing the H3 helixes of the MO25α repeats R4, R3, and R2 (Figures 1E and 2A; termed the “αC site” here). Tethered by hydrophobic and hydrogen-bonding interactions (Figure 1E), the αC-helix forms the major interaction surface, contributing a total of 405 Å^2^ buried surface area on the MO25α concave surface.

C-terminal to the αC helix, a second hydrogen-bonding network with comparable buried surface area (270 Å^2^) to the αB site is present, and involves residues Leu124, Asn126, and Tyr185 from the STRADα helix αE (Figures 1E and 2A; termed the “αE site” here). This region interacts with Glu93, Lys96, and Phe92 from the R1-H3 helix of MO25α (Figure 1E). Together, the αB site and the αE site appear to act as anchor regions, positioning the αC-helix to run along the H3 helices of R1–R5 of MO25α.

Additional interactions are found between Phe178 of MO25α, forming hydrophobic stacking interactions with residues from the N-terminal β4 and β5 strands of STRADα (termed the “β4/β5 site” here; Figures 1E and 2A). STRADα and STRADβ also possess an insertion of ten residues (221–229) in the activation loop that is not observed in TAO2 or other STE20 kinases (Figure 1A). Within this insertion, His223, Gly224, and Arg226 show weak interactions with the R0 and R1 helical repeat of MO25α (termed the “activation loop site” here; Figure 2A). This interaction perhaps explains why the STRADα activation loop is ordered. All of the key interacting interface residues are highly conserved between species of STRADα and MO25α (Figures 2 and S5).

### The MO25α Concave Surface Is Required for STRADα Binding

**Figure 3:**
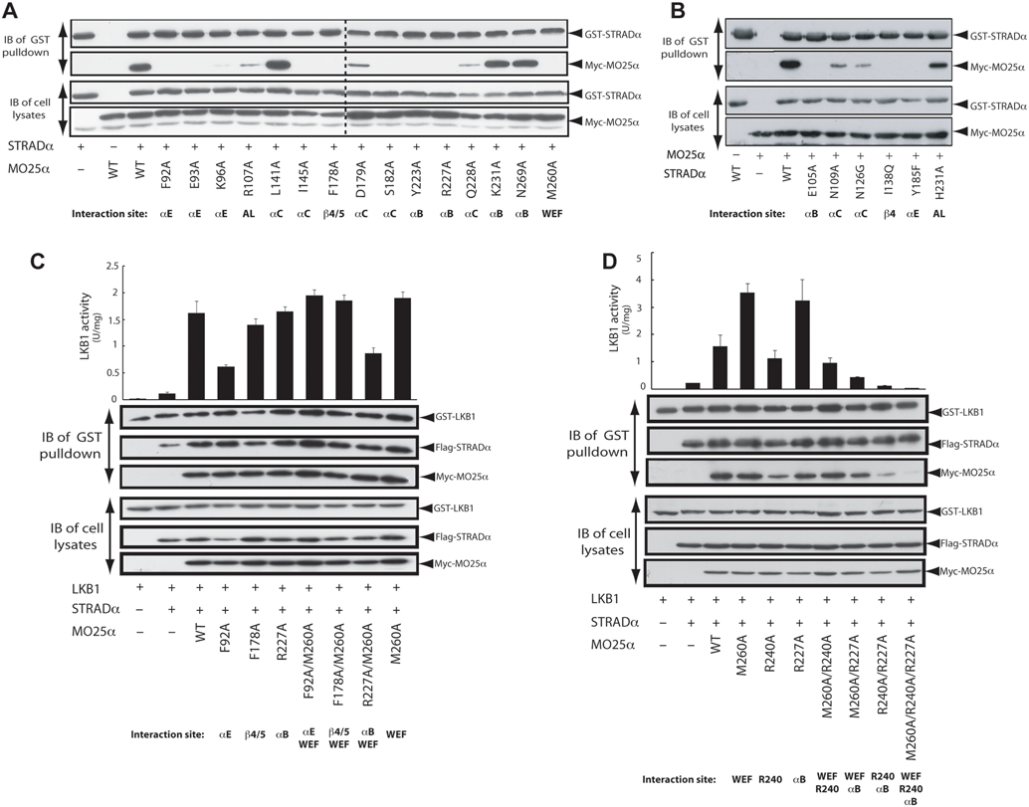
Mutation of MO25α concave surface residues abolishes STRADα and LKB1 binding. (A and B) The indicated constructs of GST-STRADα and Myc-MO25α were expressed in 293 cells. Cells 36 h post-transfection were lysed, and GST-STRADα was purified with glutathione-Sepharose. The purified GST-STRADα preparation (upper panels), as well as the cell extracts (lower panels), was immunoblotted (IB) with the indicated antibodies. Similar results were obtained in three separate experiments. Dotted line indicates the junction of two gels. AL, activation loop. (C and D) Two hundred ninety-three cells were cotransfected with the indicated constructs of GST-LKB1, Flag-STRADα, and Myc-MO25α. Cells 36 h post-transfection were lysed, and GST-LKB1 was purified and assayed for its ability to phosphorylate the LKBtide peptide. Kinase activities are representative of three independent assays carried out in triplicate (error bars represent the standard deviation for one experiment). Affinity-purified GST-LKB1 preparation (upper panel), as well as cell extracts (lower panel), was immunoblotted with the indicated antibodies.

The structure of the STRADα/MO25α complex shows that, in addition to the WEF binding pocket on the convex surface of MO25α, a major network of interactions between STRADα and the concave surface of MO25α is observed over the αB, αC, αE, β4/β5, and activation loop sites. To test the importance of these additional interactions, we investigated how mutations of residues located on the MO25α concave surface affected interaction with STRADα. We mutated residues in MO25α in the novel αB, αC, αE, β4/β5, and activation loop binding sites as well as the previously characterised WEF pocket (Figure 3A). As reported previously, mutation of Met260 in the WEF pocket of MO25α abolishes its ability to interact with STRADα in HEK293 cells [16]. However, we also observed that mutations in the two anchor regions (Phe92, Glu93, and Lys96 from the αE site and Tyr223 and Arg227 from the αB site) abolished MO25α binding to STRADα (Figure 3A). Similarly, mutating Phe178 in the β4/β5 site, Ile145 and Ser182 in the αC site, or Arg107 in the activation loop site markedly disrupted the MO25α-STRADα interaction. Mutations of Leu141, Lys231, and Asn269 in the αC site did not significantly affect binding (Figure 3A). Mutation of the reciprocal interacting residues on STRADα, including Glu105, Asn109, Asn126, Ile138, and Tyr185, also abolished or markedly reduced binding to MO25α (Figure 3B). These results confirm the importance of the network of interactions between the concave surface of MO25α and STRADα in enabling the stable association between these two proteins, at least in the absence of LKB1.

Previous work has shown that MO25α mutants in which the WEF pocket was disrupted, and that were no longer able to form a complex with STRADα, were still capable of forming a heterotrimeric complex with LKB1 and STRADα [8],[15]. Similarly, MO25α mutants in which key STRADα binding residues located within the concave surface were mutated are still capable of interacting with the LKB1/STRADα complex (Figure 3C). Even double MO25α mutants in which both the WEF pocket and the αB, αE, or β4/β5 sites were disrupted were capable of associating with the LKB1/STRADα complex (Figure 3C). Moreover, the specific activity of LKB1/STRADα complexes associated with these MO25α mutants was either normal or only moderately reduced (Figure 3C). This suggests the presence of additional interactions between MO25α and LKB1 in the presence of STRADα.

Earlier studies revealed that mutation of a conserved Arg240 residue located on the concave surface of MO25α reduced interaction with LKB1 complexed to STRADα lacking the WEF motif [15]. Arg240 might be involved in interaction with LKB1, as this residue is located on the concave surface of MO25α, distant from STRADα (Figure 2). To further investigate the role of Arg240 in enabling MO25α to associate with LKB1/STRADα, we mutated Arg240 alone or in combination with residues in either the WEF pocket (Met260) or the αB STRADα binding sites (Arg227). We found that mutation of Arg240 alone does not prevent MO25α from interacting with LKB1/STRADα (Figure 3D). However, a double MO25α mutant lacking Arg240 and a key concave surface-binding site in the αB site (Arg227), markedly impaired binding to LKB1/STRADα (Figure 3D). A triple mutant of MO25α lacking Arg240, Arg227, and the WEF pocket site failed to associate with LKB1/STRADα and stimulate LKB1 activity (Figure 3D). These observations indicate that MO25α possesses three sites with which it can interact with the LKB1/STRADα complex (Figure 2), namely two STRADα binding regions (extensive concave MO25α surface and WEF pocket) as well as a putative LKB1 binding site (Arg240).

### The STRADα/MO25α Interaction Is Similar to the CDK/Cyclin Complex

**Figure 4:**
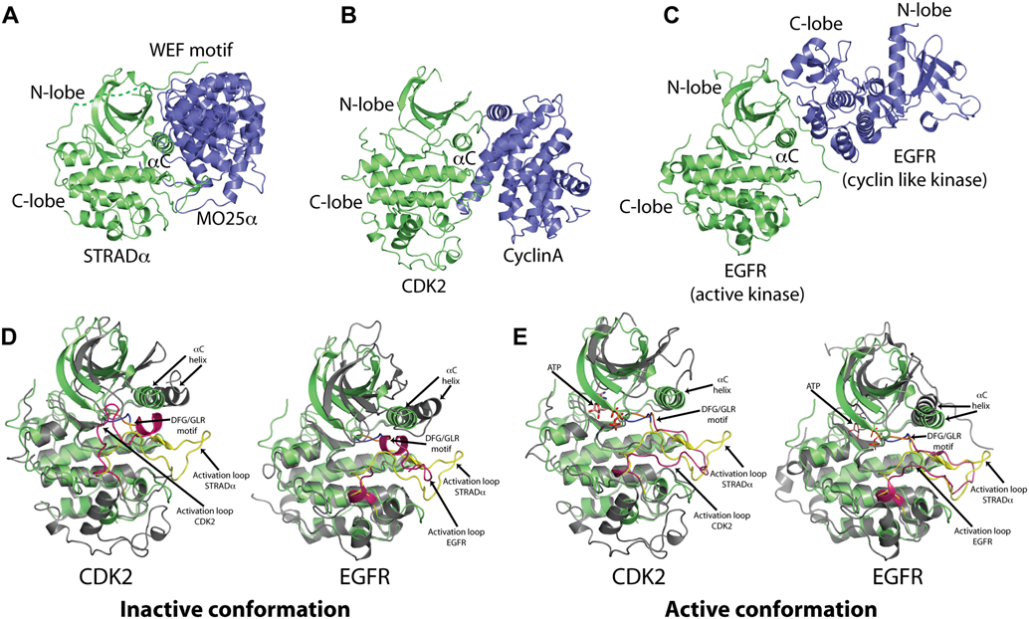
Structural comparison of the STRADα/MO25α interaction. (A–C) Resemblance of (A) STRADα/MO25α complex with (B) the CDK2/cyclin A complex (PDB ID 1FIN) and (C) the EGFR/EGFR kinase domain dimer (PDB ID 2GS2). The kinases are shown as green ribbons, with the binding partners shown as blue ribbons. The αC-helix, where the binding of the “activator” is centred, is labelled. (D and E) Comparison of the STRADα structure (green) to the active and inactive structures of CDK2 and EGFR (gray). Residues from the C-lobe of STRADα (152–431) were superimposed onto the structures of inactive CDK2 (PDB ID 1HCK [56]) and EGFR kinase (PDB ID 2GS7), and active CDK2 (PDB ID 1JST) and EGFR (PDB ID 2GS2). The activation loop of STRADα has been coloured yellow, and the activation loops of CDK2 and EGFR kinase are shown in magenta.

Inspection of the STRADα/MO25α complex reveals an unexpected resemblance to the interaction between activated cyclin-dependent kinase 2 (CDK2) and its activating regulatory subunit cyclin A (Figure 4A and 4B) [30]. Although MO25α/β isoforms are not related to cyclins at the primary sequence level, both proteins consist of multiple α-helical repeats. Crystal structures of CDK2/cyclin A complex have revealed cyclin A binds to the so-called “PSTAIRE (αC) helix” of CDK2 kinase as well as the loop immediately preceding this helix [30]. Comparisons between free CDK2 and CDK2/cyclin A complex structures have shown that the cyclin molecule orients a conserved glutamate residue (Glu51) from the αC-helix of the protein kinase to allow formation of an ion pair with a lysine residue (Lys33) from the conserved VAIK motif [30], which keeps the CDK2 kinase in a closed conformation (Figure 4B). Similarly, the position of MO25α in the STRADα/MO25α complex is centred on helix αC and the loop preceding this helix (αB region; Figure 1E). The interaction between Glu118 from the αC-helix and Arg100 from the VAIK (VTVR in STRADα) motif (analogous to the Glu51-Lys33 interaction in CDK2) is maintained, albeit via two water molecules (Figure 1B).

Another example in which this type of interaction is involved in regulating the activity of protein kinases is the ligand-induced dimerisation of the EGFR family of tyrosine kinases (Figure 4C). Although this type of dimer has not been observed in solution, crystal structures and biochemical data demonstrate the importance of dimer formation that involves the intermolecular interaction of the EGFR αC-helix on one monomer and the C-lobe on the other monomer (Figure 4C) [31]. A comparison between the structure of active, dimeric EGFR kinase with the monomeric form reveals the role of dimerisation for keeping the EGFR kinase in the closed and active conformation. Similarly, the structure of STRADα in complex with MO25α resembles the closed conformation of both CDK2 and EGFR kinase, with its activation loop and αC-helix positioned in an orientation that is typical of active protein kinases (Figure 4D and 4E). Such regulatory mechanism may also explain why some members of the EGFR family of kinases that lack kinase activity and are classified as pseudokinases (Her3) are still able to exert their function [31], despite their “inactivatory” substitutions, similar to what has been observed for STRADα (Figure 1C).

The interactions in the EGFR homodimer, the CDK2/cyclin A heterodimer, and the STRADα/MO25α complex are similar only in general topological terms. However, it appears that the mechanism of protein kinase interaction via helix αC with their activity modulators is wider than previously thought, and not exclusive to the CDK family of kinases. Indeed, there are many examples of how protein kinases are stabilised in an active conformation via helix αC. These include members of the MAP kinase family [32], the AGC family of kinases ([33]–[35], and several tyrosine kinases ([36]). Although in these examples the αC-helix is stabilised by flanking N- or C-terminal sequences/domains present in the same polypeptide chain, the mechanisms of allosteric activation are similar.

### STRADα ATP Binding Is Markedly Enhanced by MO25α

**Figure 5:**
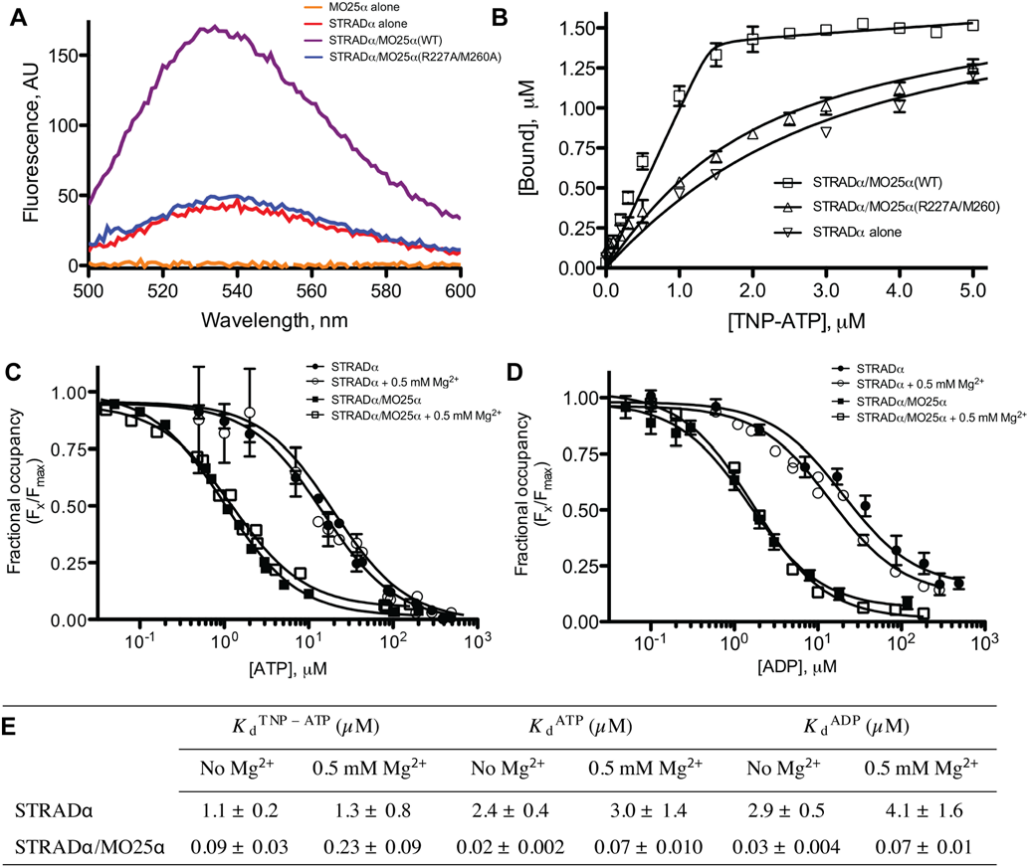
MO25α enhances the ability of STRADα to bind ATP and APD in a Mg^2+^-independent manner. (A) Fluorescence emission spectra (excitation 410 nm) of TNP-ATP (5 µM) bound to the indicated forms of STRADα (2 µM) and/or MO25α (2 µM). A reference cuvette containing TNP-ATP (5 µM) only was subtracted as background. (B) Saturation binding experiments for STRADα, STRADα complexed to MO25α (WT, wild type), and MO25α (R227A/M260A) to TNP-ATP. Bound was defined as (*F*_x_/*F*_max_) [R], where *F*_max_ and *F*_x_ are maximal and fractional fluorescence (recorded at 540 nm), respectively, and [R] equals the binding capacity, defined by the enzyme concentration, fixed at 1.5 µM. Equilibrium binding curves were then fitted to the quadratic equation suitable for tight binding interactions with ligand depletion (see Materials and Methods). Data are shown as an average of three independent experiments±SEM. (C and D) Displacement of TNP-ATP by ATP and ADP in the presence and absence of 0.5 mM MgCl_2_. Concentrations of TNP-ATP (5 µM), STRADα (2 µM), and STRADα/MO25α (2 µM) complex were fixed, and either ATP or ADP was titrated (0.05–500 µM). Emission at 540 nm was recorded, and the fractional occupancy (*F*_x_/*F*_max_) was plotted as a function of added nucleotide concentration. Dose-response curves were fitted using GraphPad-PRISM (see Materials and Methods). Data are shown as an average of three independent experiments±SEM. (E) Equilibrium binding constants for TNP-ATP, ATP, and ADP in the presence and absence of 0.5 mM MgCl_2_. *K*_d_ values were calculated as explained in Materials and Methods.

Although MO25α appears to induce a STRADα active conformation similar to CDK2/cyclin A, the effect of this “active conformation” cannot be measured through ATPase/kinase activity due to STRADα being a pseudokinase. Instead, we investigated how affinity of ATP for STRADα was modulated by its interaction with MO25α. We used the fluorescent ATP analogue 2′,3′-O-2,4,6-trinitrophenyl-ATP (TNP-ATP), whose fluorescence emission is enhanced upon its titration with ATP-binding proteins/enzymes [37], a feature that has previously been exploited to measure equilibrium binding constants of kinases for ATP [4]. Using this approach, the *K*_d_ of STRADα for TNP-ATP in the absence of MO25α was determined to be 1.1 µM (Figure 5A, 5B, and 5E). *K*_d_ values of STRADα for ATP and ADP were also assessed by their ability to displace bound TNP-ATP and found to be 2–3 µM (Figure 5C, 5D, and 5E). Strikingly, addition of an equimolar amount of MO25α to STRADα enhanced binding of TNP-ATP by an order of magnitude (Figure 5A, 5B, and 5E) and TNP-ATP displacement by two orders of magnitude (Figure 5C, 5D, and 5E), indicating significantly stronger affinity compared to the interaction of ATP as a substrate to active kinases. In contrast, the binding of STRADα to TNP-ATP was not enhanced by addition of the MO25α(R227A/M260A) mutant that is unable to bind STRADα (Figure 5A and 5B). The lack of a Mg^2+^ binding motif on STRADα suggests that Mg^2+^ should not contribute to the STRADα-ATP interaction. Indeed, Mg^2+^ did not affect binding of STRADα to TNP-ATP or displacement of TNP-ATP by ATP or ADP (Figures 5 and S6). This is in contrast with the CASK “pseudokinase,” where Mg^2+^ reportedly inhibits ATP binding and hence kinase activity [4].

It should be noted that although STRADα does not appear to require Mg^2+^ ions to bind ATP, most cellular ATP is complexed to Mg^2+^ ions. Although there is no space for Mg^2+^ to bind in the canonical protein kinase mode through the DFG motif, Mg^2+^ ions could reside in the solvent-exposed region of the phosphate moiety, replacing one of the ordered water molecules. Alternatively, it is possible that conformational changes in the structure could accommodate Mg^2+^ without affecting the ability of STRADα to bind MO25α (see below). As mentioned previously, the canonical Mg^2+^ coordinating residues appear to have been substituted through evolution with positively charged residues (Arg240 and H200), thus making redundant the role of Mg^2+^ ions.

### ATP Stimulates Binding of STRADα to MO25α

**Figure 6:**
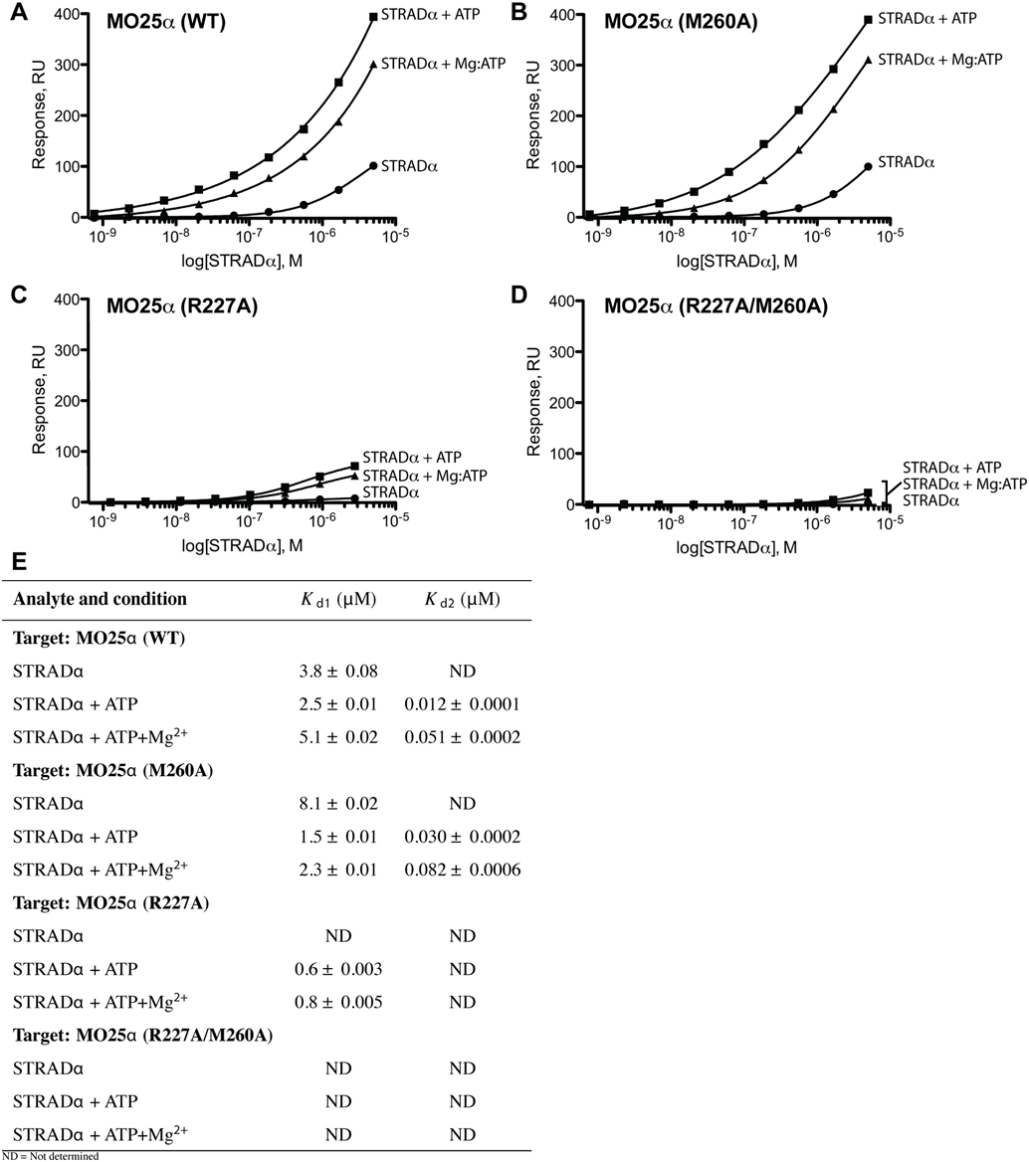
ATP enhances the ability of STRADα to bind MO25α in a Mg^2+^-independent manner. Binding of STRADα to MO25α was assessed in an SPR BIAcore assay by immobilising (A) MO25α (WT, wild type), (B) MO25α (M260A), (C) MO25α (R227A), and (D) MO25α (R227A/M260A) to a CM5 sensor chip, and STRADα was allowed to bind over 50 s by injecting different concentrations over a range of 0.4 nM to 5 µM in the presence or absence of 0.1 mM ATP and/or 1 mM MgCl_2_. Response levels for specific binding of STRADα to MO25α was plotted against STRADα concentration (log scale), using, where appropriate, a variable slope model to determine the Hill slope from the data. Similar results were obtained in at least two separate experiments. (E). Reported *K*_d_ values were calculated by measuring association (*k*_a_) and dissociation (*k*_d_) rates (Table S1) from the BIAcore sensorgram data shown in Figure S7 and using Scrubber-2 software. *K*_d_ values reported here were calculated as *K*_d_ = *k*_d_/*k*_a_ (see Materials and Methods). Equilibrium binding constants were also calculated from a saturation binding model, and similar values were obtained, as expected for specific binding that follows the law of mass action (see Figure S7 and Materials and Methods). ND = not determined.

To further investigate the functional consequences of ATP binding to STRADα, we employed quantitative SPR measurements to evaluate how ATP influenced affinity of STRADα for MO25α (Figures 6 and S7). In the absence of ATP, the binding of STRADα for MO25α was fitted to a single-site binding equation (Figure 6A and 6E). From measuring the rate constants for association and dissociation (Figure S7 and Table S1), the dissociation constant *K*_d_ was calculated as 3.8 µM (Figure 6A and 6E). However, in the presence of ATP, binding could be fitted to a two-site binding equation (Hill slope of 0.4, Figure 6A and 6E). The second binding constant (*K*_d2_) was measured as 12 nM, over two orders of magnitude higher than *K*_d1_ calculated as 2.5 µM (Figure 6A and 6E). MgATP enhanced binding of STRADα to MO25α, to a similar extent as ATP (Figure 6A). These results indicate that binding of ATP to STRADα leads to a high-affinity MO25α interaction site being exposed. Mutation of Met260 in the WEF binding pocket of MO25α did not significantly affect binding of MO25α to STRADα, nor did it influence the effect of ATP at enhancing interaction (Figure 6B and 6E). It should be noted that this observation contrasts with the data obtained from coexpression studies in 293 cells (Figure 3A) and previous studies [16], in which mutation of Met260 inhibits MO25α binding to STRADα, suggesting that the WEF pocket is required for cellular complex assembly of MO25α and STRADα. Mutation of Arg227, in the newly identified concave site of MO25α, which interacts with the αB site of STRADα, virtually abolished binding of STRADα observed by SPR in the absence of ATP. In the presence of ATP or MgATP, no two-site binding of MO25α(R227A) to STRADα was detected, displaying only low micromolar binding with a single site (Figure 6C and 6E). A double MO25α(R227A/M260A) mutant failed to interact with STRADα even in the presence of ATP (Figure 6D and 6E). These results indicate that the key STRADα high-affinity binding site on MO25α lies on the concave surface and is only recognized by STRADα in the presence of ATP. Together with the finding that MO25α also enhances affinity of STRADα for ATP (Figure 5), this suggests that the interaction of ATP and MO25α to STRADα is cooperative. A similar synergistic mechanism is observed for the PKA catalytic subunit where a nucleotide analog was shown to stabilise a complex with the PKI inhibitory peptide [38]. However, in the case of PKA/PKI interaction the γ-phosphate cannot be transferred because there is no acceptor, whereas in case of STRAD, it cannot be transferred because of the lack of a base catalyst.

### ATP and MO25α Are Required for STRADα Activation of LKB1

**Figure 7:**
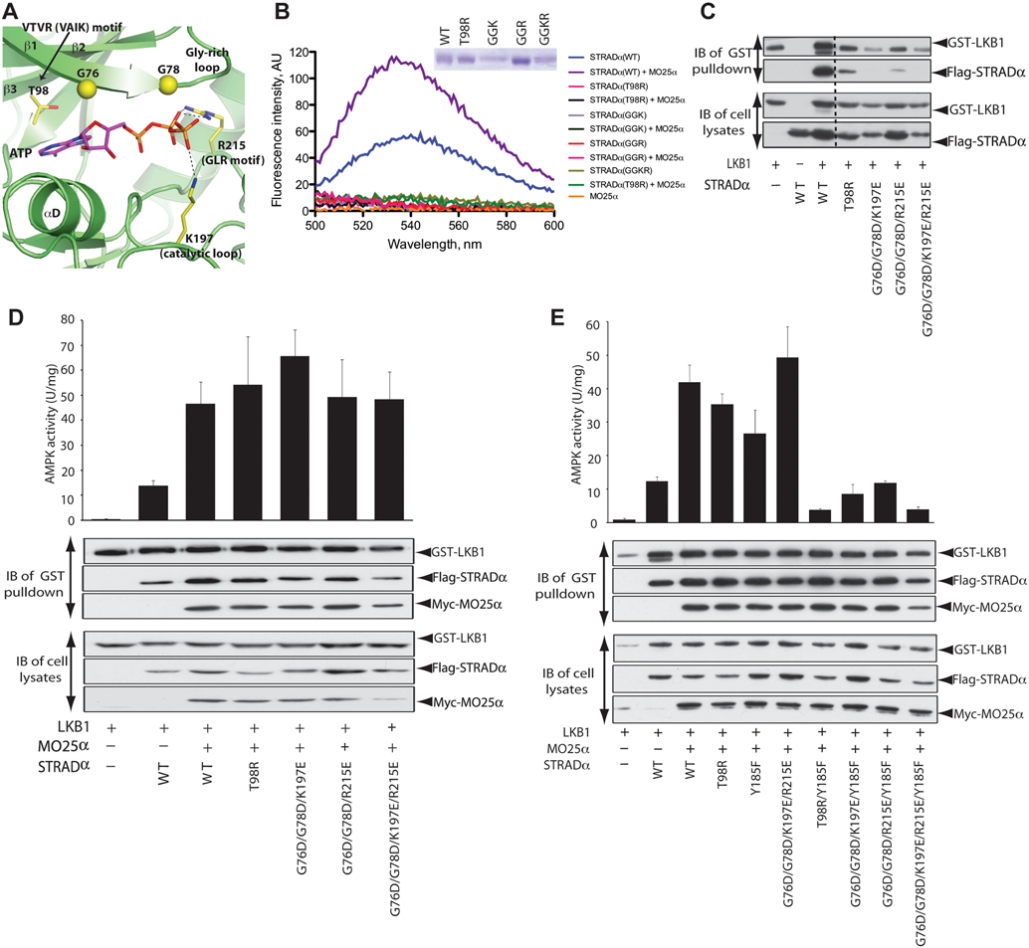
Interaction of ATP and MO25α with STRADα controls LKB1 activity. (A) The structure of the ATP binding site of STRADα in which the key interacting residues are emphasized. (B) Fluorescence emission spectra (excitation 410 nm) of TNP-ATP (5 µM) bound to wild-type and mutant forms of STRADα (2 µM) and/or wild-type MO25α (2 µM). A reference cuvette containing only TNP-ATP (5 µM) was subtracted as background. A Coomassie Blue-stained SDS-PAGE gel of each form of STRADα analysed is shown (GGK = G76D+G78D+K197E, GGR = G76D+G78D+R215E, and GGKR = G76D+G78D+K197E+R215E). (C) Wild-type GST-LKB1 and indicated forms of Flag-STRADα were expressed in 293 cells in the absence of MO25α. Cells at 36 h posttransfection were lysed and GST-LKB1 affinity purified on glutathione-Sepharose. The purified GST-LKB1 preparation (upper panel), as well as the cell extracts (lower panel), was immunoblotted (IB) with the indicated antibodies. Similar results were obtained in three separate experiments. Dotted line indicates where the gel was cut. (D and E) 293 cells were co-transfected with the indicated constructs of GST-LKB1, Flag-STRADα, and Myc-MO25α. Cells at 36 h posttransfection were lysed, and GST-LKB1 was affinity purified and assayed for the ability to activate the heterotrimeric AMPK complex expressed in *E. coli*, as described in Materials and Methods. Kinase activities are representative of three independent assays carried out in triplicate (error bars represent the SD for a single triplicate experiment). Affinity-purified GST-LKB1 preparation (upper panel), as well as cell extracts (lower panel), was immunoblotted with the indicated antibodies.

Having established that ATP increases the affinity of STRADα-MO25α interaction, we next explored whether ATP binding to STRADα also affects assembly and activity of the LKB1 heterotrimeric complex. Using the STRADα-ATP structure, a number of STRADα mutants were designed to disrupt binding of the adenine or phosphate moieties of ATP (Figure 7A). Four of these were indeed unable to interact with TNP-ATP in the presence or absence of MO25α (Figure 7B). Interestingly, these mutants also affected association with LKB1 when coexpressed in 293 cells (Figure 7C), suggesting that binding of ATP to STRADα, in the absence of MO25α, enhances the ability of STRADα to interact with LKB1. However, these mutants were capable of forming complexes with LKB1 when coexpressed with LKB1 and MO25α (Figure 7D), that retained catalytic activity as measured by activation of AMPK (Figure 7D). It is possible that binding of MO25α to these STRADα mutants compensates for their inability to bind ATP, by inducing a closed “active-like” conformation of STRADα, capable of binding and activating LKB1. To explore this idea, we generated mutants of STRADα incapable of binding to both ATP and MO25α. Strikingly, we found that these combined STRADα mutants lost their ability to activate LKB1, despite still being capable of forming a heterotrimeric complex (Figure 7E).

Taken together, these observations suggest that the closed “active-like” conformation of STRADα is maintained through binding to ATP and/or MO25α, and is required for activation of LKB1. Mutations that prevent STRADα from binding to ATP or MO25α do not affect activation of LKB1 (Figures 3 and 7), suggesting that ATP binding to STRADα can compensate for loss of MO25α interaction and vice versa. However, loss of both ATP and MO25α binding prevents STRADα from activating LKB1. Such mutations may leave STRADα in the open “inactive-like” conformation incapable of activating LKB1. We have tried unsuccessfully to crystallise STRADα in the absence of MO25α in order to demonstrate this. Binding of ATP to several kinases, including the EGF receptor tyrosine kinase, promotes the closed, active conformation of these enzymes. Moreover, as discussed above, binding of cyclin to CDK2 is reminiscent of the interaction of STRADα with MO25α, and interaction of cyclin A is well known to promote the closed active conformation of CDK2 [30].

**Figure 8:**
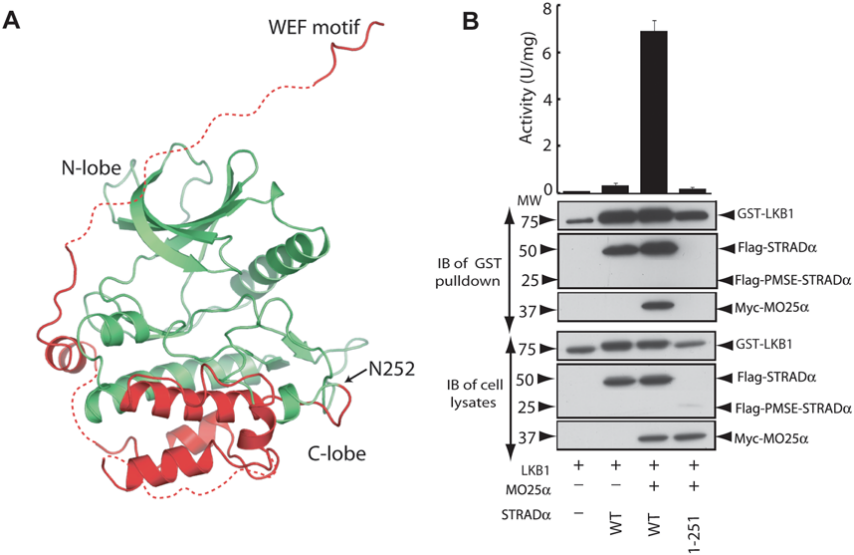
PMSE truncation and the stability of STRADα. (A) Structure of STRADα in which the region beyond Asn252 that is truncated in PMSE patients is coloured in red. (B) A total of 293 cells were cotransfected with the constructs encoding wild-type GST-LKB1 and Myc-MO25α together with constructs encoding wild-type or PMSE mutant Flag-STRADα. Cells at 36 h posttransfection were lysed, and GST-LKB1 was affinity purified and assayed for ability to phosphorylate the LKBtide peptide. Kinase activities are representative of three independent assays carried out in triplicate (error bars represent the SD for a single experiment carried out in triplicate). Affinity-purified GST-LKB1 preparation (upper panel), as well as cell extracts (lower panel), was immunoblotted (IB) with the indicated antibodies.

### The PMSE Mutation Structurally Impairs STRADα

The PMSE-causing mutation in humans results in a STRADα truncation at residue 251, thus removing the last 180 amino acids [14]. Inspection of the STRADα structure reveals that this mutation would delete almost half of the C-terminal lobe of the pseudokinase domain, beginning with structurally vital components such as helix αF (Figure 8A). This could destabilize the STRADα protein, as helix αF forms numerous hydrophobic interactions within the C-lobe of the pseudokinase domain, which would become solvent exposed in the PMSE mutant. We attempted to express the PMSE-STRADα (residues 1–251) mutant in 293 cells and found that it was expressed at significantly lower levels than full-length STRADα (Figure 8B), consistent with this fragment being unstable. Moreover, STRADα (1–251) failed to interact with or activate LKB1 (Figure 8B). These results confirm that the STRADα mutation found in PMSE patients represents a loss-of-function mutation that would be unable to stimulate the LKB1 pathway. This could account for the elevated mTOR pathway activity that was observed in neuronal cells derived from PMSE patients [14].

### Concluding Remarks

We have described the first structure of the STRADα pseudokinase and its interaction with MO25α, a heterodimeric interaction within the heterotrimer LKB1 tumour suppressor complex. A key discovery is the identification of an unexpected extensive interaction between STRADα and the concave surface of MO25α, previously proposed to harbour a ligand binding site [16]. Armadillo repeat proteins that are structurally related to MO25α, such as PUM1 [19], β-catenin [17], and importin-α [18], also bind their macromolecular partners along their concave surface. In general topological terms, the STRADα/MO25α complex resembles the interaction between CDK2 and cyclin A, and the EGFR/EGFR dimer, and provides another example of protein kinase regulatory mechanism via helix αC.

**Figure 9:**
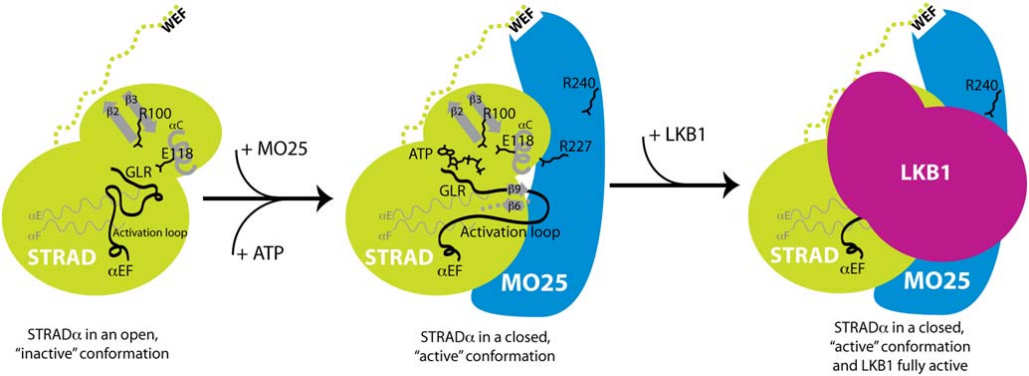
Model of how STRADα/MO25α might interact and activate LKB1. The model is based on known mutagenesis and structural data discussed in this paper. Binding of either ATP and/or MO25α to STRADα induces STRADα to adopt a closed conformation, leading to the assembly of a fully active LKB1 complex.

Our data show that, despite lacking most essential catalytic residues, STRADα has maintained its ability to adopt a closed active-like conformation, which binds ATP and possesses an ordered activation loop similar to active protein kinases. This closed conformation is stabilized through binding of ATP and/or MO25α. Moreover, binding of MO25α to STRADα markedly enhances affinity for ATP, and binding of ATP to STRADα stimulates interaction with MO25α. Our findings support a model in which binding of either MO25α or ATP is sufficient to enable STRADα to activate LKB1. Consistent with this, mutant forms of STRADα that are incapable of binding both ATP and MO25α can no longer activate LKB1, whereas mutant forms of STRADα that retain the ability to bind either ATP or MO25α still activate LKB1. Thus, the closed active-like conformation, rather than catalytic phosphoryl transfer activity, is likely to be the key to the mechanism by which STRADα activates the LKB1 tumour suppressor. A model of how STRADα/MO25α might interact and activate LKB1 based on known mutagenesis and structural data is presented in Figure 9. Future work may establish other examples of pseudokinases that, like STRADα, regulate signal transduction networks through their conformational state alone.

Very recent reports have described the structures of VRK3 [39] and ROP2 [40] pseudokinases, both incapable of binding ATP. Both studies support the notion put forward in this paper that pseudokinases may function by means of conformational state rather than catalytic activity, although in an ATP-independent manner.

## Materials and Methods

### General Methods and Buffers

Restriction enzyme digests, DNA ligations, and other recombinant DNA procedures were performed using standard protocols. All mutagenesis were performed using the QuickChange site-directed mutagenesis method (Stratagene) with the KOD polymerase (Novagen). DNA constructs used for transfection were purified from *E. coli* DH5α using Qiagen Plasmid kits according to the manufacturer's protocol. All DNA constructs were verified by DNA sequencing, which was performed by the Sequencing Service, College of Life Sciences, University of Dundee, United Kingdom, using DYEnamic ET terminator chemistry (Amersham Biosciences) on Applied Biosystems automated DNA sequencers. Lysis buffer used for HEK 293 cells was 50 mM Tris-HCl (pH 7.5), 1 mM EGTA, 1 mM EDTA, 1% (w/v) Nonidet P40 (substitute), 1 mM sodium orthovanadate, 50 mM sodium fluoride, 5 mM sodium pyrophosphate, 0.27 M sucrose, 0.1% (v/v) 2-mercaptoethanol, 1 mM benzamidine, and 0.1 mM PMSF. Buffer A was 50 mM Tris-HCl (pH 7.5), 0.1 mM EGTA, and 0.1% (v/v) 2-mercaptoethanol. SDS sample buffer contained 50 mM Tris-HCl (pH 6.8), 2% (w/v) SDS, 10% (v/v) glycerol, 0.005% (w/v) bromophenol blue, and 1% (v/v) 2-mercaptoethanol. TBS-T buffer was Tris-HCl (pH 7.5), 0.15 M NaCl, and 0.5% (v/v) Tween. All protein concentrations were determined using the Bradford reagent (Bio-Rad) and by measuring the absorbance at 595 nm, unless indicated otherwise.

### Cloning, Protein Expression, and Purification

A bicistronic expression system was used to coexpress and purify the STRADα/MO25α complex in *E. coli*. Expression vectors were kindly donated by Dr. Roger Williams (University of Cambridge, United Kingdom). The cloning procedure was followed as described in [41]. Briefly, both STRADα and MO25α genes were subcloned as separate cassettes from the pOPT single vectors into a pOPCH polycistronic vector. Full-length MO25α (residues 1–341) was subcloned from a pOPT (no tag) vector as an NdeI/BamH1 insert. STRADα (residues 59–431) with an N-terminal 6-His tag followed by a Tobacco Etch Virus (TEV) protease site (sequence MAHHHHHHMENLYFQG) was subcloned from a POPTH vector as a BspE1/Mlu1 insert. For more information on the expression and purification of STRADα for activity assays, see Text S1.

N-terminally 6-His-tagged STRADα was coexpressed with untagged full-length MO25α in *E. coli* BL21(DE3)pLysS cells. Cells were grown in Luria Bertani medium to A_600_ = 0.7 at 37°C, before protein expression was induced by the addition of 250 µM isopropyl-β-d-thiogalactopyranoside (IPTG) and incubated for a further 16 h at 26°C. Cells were harvested by centrifugation for 30 min at 3,500*g* and resuspended in ice-cold lysis buffer (50 mM Tris-HCl [pH 7.8], 50 mM NaCl, 10% glycerol, 20 mM imidazole, 1 mM benzamidine, 0.2 mM EGTA, 0.2 mM EDTA, 0.1 mM PMSF, 0.075% (v/v) β-mercaptoethanol, 0.5 mg/ml lysozyme, and 0.3 mg/ml DNAse-I. Cells were lysed using a French Press cell disrupter (18,000 psi), and the lysate was cleared by centrifugation at 26,000*g* for 30 min. The supernatant was then passed through a 0.22-µm filter before loading onto a 5-ml HiTrap IMAC HP column (GE Healthcare) previously charged with Ni^2+^. The column was then washed with ten volumes of wash buffer (lysis buffer without lysozyme, DNAse-I, and PMSF), and the STRADα/MO25α complex was eluted by applying a gradient of 20–300 mM imidazole in wash buffer. The sample was then concentrated to 3 ml and loaded onto a Superdex 75 26/60 gel filtration column, pre-equilibrated in 25 mM Tris (pH 7.8) and 1 mM DTT. For the methylated protein complex, the sample was dialyzed into 25 mM Tris-HCl (pH 7.5), 50 mM NaCl, 10% glycerol, 1 mM benzamidine, and 0.075% (v/v) β-mercaptoethanol after imidazole elution, and subjected to lysine methylation using formaldehyde and dimethylamine-borane complex, as described elsewhere [21]. The methylated STRADα/MO25α complex was then passed through a desalting column prior to loading onto a gel filtration column as explained above. The binary complex eluted as a single peak, and its purity was assessed by SDS-PAGE.

### Crystallization, Structure Solution, and Refinement

The STRADα/MO25α complex was concentrated to 7.5 mg/ml, followed by addition of ATP to a final concentration of 10 mM and MgCl_2_ (final concentration of 1 mM). The sitting drop vapour diffusion method was used to grow crystals by mixing 1 µl of protein solution, 1 µl of mother liquor. For the unmethylated complex, the optimised mother liquor consisted of 20 mM Li_2_SO_4_, 50 mM sodium citrate (pH 5.6), 6% (v/v) PEG4000. For the methylated complex, the mother liquor was composed of 0.1 M MES (pH 6.4), 10% (v/v) PEG8000. For both conditions, 0.25 µl of 1 M NDSB-256 was added to the crystallisation drop. Rod-shaped crystals of the unmethylated complex appeared after 3 h and grew to 0.05 mm (maximum dimension) after 24 h. The methylated sample yielded bigger crystals that appeared after 24 h and grew to a maximum length of 0.5 mm after 3 d. Crystals were flash frozen in liquid nitrogen after cryoprotection with mother liquor containing 20% (v/v) glycerol (unmethylated) and 25% (v/v) PEG8000 and 10% (v/v) PEG300 (methylated).

Data were collected at 100 K on stations ID14-3, ID14-4, and ID23-2 at the European Synchrotron Radiation Facility (ESRF) and processed using the MOSFLM and SCALA programs from the CCP4 package [42] (Table 1). The structures of the unmethylated/methylated complexes were solved by a combination of molecular replacement with MOLREP [43] and real-space searches with FFFEAR [44]. An initial molecular replacement run was carried out with MOLREP using the 1.85 Å structure (Protein Data Bank ID [PDB ID] 1UPK) of MO25α [16] as a search model. Using the resulting phases, the STRADα molecule was then located by performing a real-space search with FFFEAR [44] using the 2.1 Å structure (PDB ID 1U5R) of TAO2 [23]. Thus, a solution with one complex in the asymmetric unit was found, and the structure was refined by alternating rounds of refinement with REFMAC5 [45] (including TLS refinement during the last macrocycles) and manual model building with the program COOT [46]. For the methylated complex, this resulted in a final model with an *R*-factor of 0.206 (*R*_free_ = 0.254) that was validated using PROCHECK [47] and MOLPROBITY [48] (Table 1). STRADα residues 292–347, 383–385, and 402–424, and MO25α residues 337–341 were not associated with clear electron density and were not included in the model.

Figures were prepared using the PyMOL molecular graphics system available at http://www.pymol.org [49]. Secondary structure was analysed using DSSP [50] and sequence alignments were performed using MUSCLE [51], which were edited and displayed using the program ALINE developed by Charlie Bond and Alexander Schüttelkopf.

### Cell Culture, Transfections, and Lysis

Two hundred ninety-three cells were cultured on 10-cm diameter dishes in 10 ml of DMEM supplemented with 10% (v/v) fetal bovine serum, 2 mM l-glutamine, 100 U/ml penicillin, and 0.1 mg/ml streptomycin. For transfection experiments, 3–9 µg of DNA were mixed with 20 µl of 1 mg/ml polyethylenimine (Polysciences) in 1 ml of plain DMEM for each dish; the mixture was left to stand for 30 min and added onto the cells. Cells were lysed 36 h posttransfection in 1 ml of ice-cold lysis buffer per dish. The cell lysates were clarified by centrifugation at 20,000*g* for 15 min at 4°C, and the supernatants divided into aliquots, frozen in liquid nitrogen, and stored at −20°C.

### Expression of Fusion Proteins in HEK293 Cells and Affinity Purification

10-cm diameter dishes of 293 cells were transiently transfected with 3 µg of the pEBG-2Tconstructs together with 3 µg of the indicated pCMV5 constructs as described above. Cells were harvested and lysed 36-h posttransfection, and the clarified lysates were incubated for 1 h on a rotating platform with glutathione-Sepharose (GE Healthcare; 20 µl/dish of lysate) previously equilibrated in lysis buffer. The beads were washed twice with lysis buffer containing 150 mM NaCl and twice with 50 mM Tris HCl, pH 7.5. For immunoblotting analysis, the beads were resuspended in SDS sample buffer after this step and the samples immunoblotted as described above. For protein kinase assays and gel electrophoresis, the beads were washed twice more with Buffer A, and the proteins were eluted from the resin by incubation with the same buffer containing 270 mM sucrose and 20 mM of reduced glutathione. The beads were then removed by filtration through a 0.44-µm filter, and the eluate was divided into aliquots and stored at −80°C.

### Assaying LKB1 by Measuring Phosphorylation of the LKBtide Peptide

The activity of recombinant LKB1/STRADα/MO25α complexes was assayed towards the LKBtide peptide substrate. All assays were performed by using 0.35 µg of recombinant proteins expressed and purified from HEK293 cells as described above. Phosphotransferase activity towards the LKBtide peptide (SNLYHQGKFLQTFCGSPLYRRR) [52] was measured in a total assay volume of 50 µl consisting of 50 mM Tris-HCl (pH 7.5), 0.1 mM EGTA, 0.1% (v/v) 2-mercaptoethanol, 10 mM magnesium acetate, 0.1 mM [γ-^32^P]ATP (200 cpm/pmol), and 0.2 mM LKBtide peptide. The assays were carried out at 30°C and were terminated after 15 min by applying 40 µl of the reaction mixture onto P81 membranes. These were washed in phosphoric acid, and the incorporated radioactivity was measured by scintillation counting as described previously for MAP kinase [53]. One unit (U) of activity represents the incorporation to the substrate of 1 nmol of γ-^32^P per minute.

### Assaying LKB1 by Measuring Activation of the Heterotrimeric AMPK Kinase

The AMPK heterotrimeric complex was purified from *E. coli*, and the AMPK activity was measured following its phosphorylation with LKB1 as reported by Lizcano et al. [52]; 10 µg of AMPK complex (α_1_β_2_γ_1_ subunits) was incubated with or without 0.3 ng of wild-type or mutant LKB1/STRADα/MO25α complex in Buffer A containing 5 mM magnesium acetate and 0.1 mM cold ATP, in a final volume of 20 µl. After incubation at 30°C for 30 min, the AMPK kinase activity was determined by adding 30 µl of 5 mM magnesium acetate, 0.1 mM [γ-^32^P]ATP (300 cpm/pmol), and 0.2 mM AMARA peptide (AMARAASAAALARRR) [54] as substrate. After incubation for 20 min at 30°C, incorporation of γ-^32^P into the peptide substrate was determined by applying the reaction mixture onto P81 phosphocellulose paper and scintillation counting as described in the previous section. One unit (U) of activity represents the incorporation to the substrate of 1 nmol of γ-^32^P per minute.

### Immunoblotting

The indicated amounts of cell lysates or purified proteins were subjected to SDS-PAGE and transferred to nitrocellulose membranes. The membranes were blocked for 1 h in TBS-T buffer containing 5% (w/v) skimmed milk. The anti-GST, anti-Flag, and anti-Myc antibodies (Sigma) were diluted 1,000-fold before the membranes were immunoblotted in the same buffer containing the indicated antibodies, for 16 h at 4°C. Membranes were then washed six times with TBS-T buffer and incubated with the appropriate horseradish peroxidase-conjugated secondary antibodies (Pierce) in TBS-T buffer containing 10% (w/v) skimmed milk. After repeating the washing steps, detection was performed using the enhanced chemiluminescence reagent (Amersham Pharmacia Biotech), and the films were developed using a film automatic processor (SRX-101; Konica Minolta Medical).

### Protein Expression for Nucleotide Binding and SPR Measurements

For nucleotide binding experiments, wild-type and mutant forms of STRADα (residues 54–431) and MO25α (residues 1–341) were expressed individually as GST fusion proteins in *E. coli*. Cells were grown in Luria Bertani medium to A_600_ = 0.7 at 37°C, and protein expression was induced by the addition of 250 µM IPTG and incubated for a further 16 h at 26°C. Cells were harvested by centrifugation for 30 min at 3,500*g* and resuspended in ice-cold wash buffer (50 mM Tris-HCl (pH 7.8), 150 mM NaCl, 5% (v/v) glycerol, 1 mM benzamidine, 1 mM EGTA, 1 mM EDTA, 0.1 mM PMSF, and 0.01% (v/v) β-mercaptoethanol, supplemented with 0.5 mg/ml lysozyme and 0.3 mg/ml DNAse-I. Cells were lysed by sonication (10 × 10 s pulses) and clarified lysates (by centrifugation at 26,000*g*) were incubated for 1 h on a rotating platform with glutathione-Sepharose (GE Healthcare; 0.5 ml/l of culture) pre-equilibrated in wash buffer. The beads were then washed with ten column volumes of wash buffer and a further 50 column volumes of high-salt wash buffer containing 500 mM NaCl. Beads were re-equilibrated in ten column volumes of wash buffer, and the proteins were eluted by incubating with PreScission protease for 16 h. Protein eluates were dialysed for 16 h against 5 l of assay buffer containing 50 mM Tris-HCl (pH 7.8), 50 mM NaCl, 270 mM sucrose, and 1 mM DTT, concentrated to 7 mg/ml, divided into aliquots, and stored at −80°C.

For SPR measurements, wild-type and mutant forms of MO25α were expressed and purified as above. His-STRADα (residues 59–431) was isolated in complex with MO25α as described for crystallisation. After gel filtration (GF) in GF buffer containing 50 mM Tris-HCl (pH 7.8), 50 mM NaCl, 270 mM sucrose, and 0.075% (v/v) β-mercaptoethanol, the STRADα/MO25α complex (20 mg) was resuspended in 20 ml of binding buffer (BB), consisting of GF buffer with increased NaCl concentration (300 mM). This sample was passed through 2 ml of Ni^2+^-agarose beads (Invitrogen), equilibrated in BB, and the beads were washed with 50 column volumes of BB containing 500 mM NaCl and were re-equilibrated with ten column volumes of BB. His-STRADα was eluted in binding buffer supplemented with 150 mM imidazole. The eluted His-STRADα sample was equally divided and dialyzed against the assay buffer mentioned above. Untagged STRADα was obtained by incubation with TEV protease for 16 h at 4°C. Uncleaved STRADα and the TEV protease were removed by passing the postcleavage sample through Ni^2+^-agarose beads. His-STRADα and untagged STRADα were finally dialyzed into assay buffer, concentrated, and stored as above. Protein concentrations were determined by measuring the absorbance of the purified proteins at 280 nm in assay buffer.

### Nucleotide Binding Assays

Fluorescent measurements of TNP-ATP (Molecular Probes), were obtained at 25°C in assay buffer (with the addition of 0.5–1.0 mM MgCl_2_ where indicated) using 1-cm pathlength cuvettes in a VARIAN Cary Eclipse Fluorescence spectrophotometer (Varian). Fluorescence was recorded using a 410-nm/540-nm excitation/emission wavelengths from 500 to 600 nm. In all cases, signal from the TNP-ATP buffer control was subtracted as background. For all binding studies, STRADα and STRADα mutants were assayed at 2 µM. In cases where STRADα/MO25α complexes were assayed, wild-type or mutant MO25α (2 µM) were preincubated for at least 2 h at 4°C prior to a fluorescence binding experiment. For saturation binding experiments, concentrated stocks of TNP-ATP were added stepwise, covering a range of concentrations from 0.05 to 30 µM. For displacement experiments, the concentration of TNP-ATP was fixed at 5 µM, and ATP or ADP was titrated in, covering a range of concentrations from 0.05 to 500 µM. In all assays, concentrated stocks of nucleotides were added to 1 ml of reaction mixture in steps of 0.5 to 1.0 µl, ensuring that the total added volume did not exceed 1% of the total volume of the reaction.

All data were analysed using GraphPad-PRISM software (http://www.graphpad.com). To calculate the *K*_d_ values for TNP-ATP, data from saturation binding experiments were fitted to the following quadratic equation suitable for tight binding interactions with ligand depletion [55]:
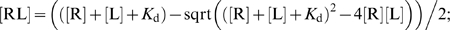
where [RL] equals the concentration of receptor/ligand complex, calculated as the fractional occupancy (*F*_x_/*F*_max_) × [R]; [R] equals the total binding capacity, fixed at 1.5 µM; and [L] equals the concentration of added TNP-ATP. In the displacement studies, equilibrium constant values for ATP and ADP were calculated by first determining the logEC_50_ value, using a standard dose-response equation: *F*_x_/*F*_max_ = minimum+(maximum−minimum)/(1+10^([N]−logEC^_50_^)^), where [N] equals the concentration of added nucleotide, and *F*_x_/*F*_max_ represents the fractional occupancy. Equilibrium constants for the competing ATP and ADP (*K*_d_^N^), were fitted using the equation: logEC_50_ = log(10^log*K*^_d_^N^ × (1+[TNP−ATP]/*K*_d_^TNP−ATP^)).

### SPR Measurements of STRADα Binding to MO25α

SPR measurements were performed using a BIAcore T100 instrument. Wild-type and mutant forms of MO25α were immobilized on a CM5 sensor chip using standard amine-coupling chemistry, and 10 mM HBS (pH 7.4) was used as the running buffer. The carboxymethyl dextran surface was activated with a 7-min injection of a 1∶1 ratio of 0.4 M 1-ethyl-3-(3-dimethylaminopropyl) carbodiimide hydrochloride (EDC)/0.1 M N-hydroxy succinimide (NHS). MO25α (5–7 µM) was coupled to the surface with a 1-min injection of protein diluted in 10 mM sodium acetate (pH 5.5). Remaining activated groups were blocked with a 7-min injection of 1 M ethanolamine (pH 8.5). MO25α was immobilised on three flow cells of a CM5 chip at densities 1,700–2,500 RU performed at 25°C, leaving one flow cell as a reference to subtract any possible nonspecific binding.

STRADα was prepared in running buffer containing 50 mM Tris (pH 7.8), 50 mM NaCl, 270 mM sucrose, 1 mM DTT, 0.005% P20, and 0.1 mg/ml BSA in the presence/absence of 100 µM ATP and 1 mM MgCl_2_, and injected over all four surfaces at nine concentrations of a 3-fold concentration series (5 µM to 0.3 nM). Each concentration was injected in duplicate over all surfaces. Association was measured for 60 s at a flow rate of 50 µl/min, and dissociation was measured for 3 min. STRADα dissociated completely from the MO25α surfaces, thus eliminating the need for a regeneration step.

Data were analysed using Scrubber 2 (BioLogic Software) and CLAMP software. Data were double referenced to the reference surface to subtract any possible nonspecific binding and to the blank buffer injections to subtract drift of the target from surface. Data were fitted to a 1∶1 or 2∶1 binding site model where appropriate. Kinetic association (*k*_a_) and dissociation rate (*k*_d_) constants were separately determined from the BIAcore sensorgrams, and equilibrium dissociation constants (*K*_d_) were calculated as: *K*_d1_ = *k*_d1_/*k*_a1_ and *K*_d2_ = *k*_d2_/*k*_a2_. Equilibrium constants were also independently calculated from a saturation binding curve, by fitting the measured response (*R*) from specific binding to the following equation: *R* = (*R*_max1_[STRAD]/([STRAD]+*K*_d1_))+(*R*_max2_[STRAD]/([STRAD]+*K*_d2_)), where *R*_max1_ and *R*_max2_ are the relative maximal changes in response for sites 1 and 2, respectively, and *K*_d1_ and *K*_d2_ are the equilibrium dissociation constants for sites 1 and 2, respectively. Dose-response curves for calculating the Hill slope (H) of the data were fitted with the following equation: *R* = minimum+(maximum−minimum)/(1+10^((logEC50−[STRAD])×H)^) using GraphPad-PRISM software.

### Accession Numbers

Coordinates and observed structure factor amplitudes have been deposited at the Worldwide Protein Data Bank (wwPDB, http://www.wwpdb.org/), with accession code 3GNI.

## Supporting Information

Figure S1 **Isolation of the heterodimeric STRADα/MO25α complex.** (A) Gel filtration profiles of His-STRADα/MO25α coexpressed in *E. coli* and crystallised in this study. The elution profile of separately expressed MO25α monomer as well as the molecular mass standards aldolase (158 kDa), conalbumin (75 kDa), ovalbumin (43 kDa), carbonic anhydrase (29 kDa), and ribonuclease A (13.7 kDa) are also shown. (B) We analysed, by SDS-PAGE, the fractions in which STRADα/MO25α dimer and MO25α monomer were eluted and stained with Coomassie Blue. There is no evidence for large molecular weight aggregates of His-STRADα/MO25α. In the His-STRADα/MO25α purification, a minor low molecular weight eluting shoulder to the main peak was found to consist of mainly uncomplexed His-STRADα. Because His-STRADα was the subunit used for nickel affinity purification of the complex, it will be expected to be present in excess.
(1.99 MB TIF)

Figure S2 **Attempts at reactivating the STRADα pseudokinase.** The indicated STRADα (residues 59–431) active site mutants were expressed in *E. coli* and tested for kinase activity in the presence of 0.2 mM γ-^32^P-ATP and 10 mM magnesium acetate, (A) alone or (B) in the presence of MO25α. Similarly, in (C) and (D), the same mutations were tested in the absence of magnesium acetate. (E–H) STRADα active site mutants were combined with mutations/deletions from the P+1 site of the kinase. (E and F) were tested in the presence of magnesium acetate, whereas (G and H) were tested in the absence of Mg^2+^. In all cases, PKA assayed in the presence of Mg^2+^ was included as a positive control. (5X = T98A+R100K+G213D+L214F+R215G)
(3.73 MB TIF)

Figure S3 **Characterisation of the MO25α PFPF motif, the STRADα WEF motif, and effects on LKB1 binding.** (A) Comparison of STRADα WEF motif, binding to the MO25α WEF pocket. WEF motifs from the STRADα/MO25α complex structure and MO25α/peptide complex determined previously by Milburn et al. [16], are superimposed (RMSD = 0.3 Å over 35 atoms) and shown as stick models with green and yellow carbon atoms, respectively. Electron density maps (*F*_o_-*F*_c_ are shown for the WEF motif determined in this study and contoured at 2.5σ). (B) The PFPF motif of MO25α binds to a STRADα hydrophobic pocket, near the ATP binding site. Electron density maps are displayed as described above. (C) The indicated constructs of GST-STRADα and untagged MO25α were expressed in 293 cells. Cells were lysed 36 h posttransfection and GST-STRADα was affinity purified on glutathione-Sepharose. The purified GST-STRADα preparation (upper panels), as well as the cell extracts (lower panel), was immunoblotted with the indicated antibodies. STRADα R227A mutant, unable to bind MO25α, was used as a control. (D) Wild-type GST-LKB1 and indicated forms of Flag-STRADα and untagged MO25α were cotransfected in 293 cells. Cells 36 h posttransfection were lysed, and GST-LKB1 was affinity purified on glutathione-Sepharose. The purified GST-LKB1 preparations (upper panels), as well as the cell extracts (lower panel), were immunoblotted with the indicated antibodies. (E) Either 0.5 or 1.0 mg of the indicated cell lysates were incubated with 5 µg of the indicated biotinylated peptides conjugated to Streptavidin-Sepharose. Following isolation and washing of the beads, the samples were subjected to SDS-polyacrylamide gel electrophoresis and immunoblotted with the indicated antibodies. (F) Activation of the bacterially expressed AMPK complex using wild-type or mutant LKB1/STRADα/MO25α complex. The purity of LKB1 complexes was analyzed by SDS-PAGE and colloidal blue staining.
(3.82 MB TIF)

Figure S4 **His-tagged STRADα and untagged STRADα bind MO25α with similar affinity.** His-STRADα was treated in the presence or absence of His-TEV protease to remove the 6-His purification tag and then repurified using nickel agarose to remove His-TEV and any uncleaved His-STRADα (see Materials and Methods). Binding was assessed by SPR analyses where (A) MO25α(WT) (wild type), (B) MO25α(M260A), (C) MO25α(R227A), and (D) MO25α(R227A/M260A) were immobilised to a CM5 sensor chip. Equivalent concentrations of His-STRADα or untagged STRADα, were allowed to bind over 50 s by injecting different concentrations over a range of 0.4 nM to 5 µM, in the presence of 0.1 mM ATP and 1 mM MgCl_2_. Response level for specific binding of STRADα to MO25α was plotted against STRADα concentration (log scale), using a variable slope model (where appropriate) to determine the Hill slope from the data. (E). Reported *K*_d_ values were calculated by measuring association (*k*_a_) and dissociation (*k*_d_) rates from the BIAcore sensorgram data shown in Figure S7 and Table S1, using Scrubber-2 software. *K*_d_ values reported here were calculated as *K*_d_ = *k*_d_/*k*_a_ (see Materials and Methods). Equilibrium binding constants were also calculated from a saturation binding model, and similar values were obtained. (see Figure S7 and Materials and Methods).
(0.66 MB TIF)

Figure S5 **Sequence conservation of STRADα and MO25α.** Sequence alignment (dark blue = conserved, white = not conserved) of STRADα (A) and MO25α (B) of the indicated species. Alignments were performed with MUSCLE and edited and displayed using ALINE (Charlie Bond and Alex Schüttelkopf). A graph of residues involved in STRADα/MO25α interaction against their contact area (green bars), is displayed. Height of the bar represents the contact area (atom pairs closer than 3.9 Å, analysed by CONTACT from the CCP4 package), divided by the molecular weight of the participating amino acid. Key STRADα catalytic motifs and the WEF motif are boxed. The secondary structure (analysed by DSSP) is shown in red. Dotted lines represent residues missing in our structural model.
(1.93 MB TIF)

Figure S6 **Binding of STRADα/MO25α complex to the ATP fluorescent analog TNP-ATP±MgCl_2_.** Saturation binding experiments for STRADα/MO25α complex to TNP-ATP in the presence/absence of 0.5 mM and 1 mM MgCl_2_. Bound was defined as (*F*_x_/*F*_max_)[R], where *F*_max_ and *F*_x_ are maximal and fractional fluorescence (recorded at 540 nm), respectively, and [R] equals the binding capacity, defined by the enzyme concentration, fixed at 1.5 µM. Equilibrium binding curves were then fitted to the quadratic equation suitable for tight binding interactions with ligand depletion (see Materials and Methods). *K*_d_ values were calculated as: 0.09±0.03 µM, 0.23±0.06 µM, and 0.09±0.04 µM for TNP-ATP, TNP-ATP+0.5 mM MgCl_2_, and TNP-ATP+1.0 mM MgCl_2_, respectively. Data shown are the average of two independent experiments.
(0.30 MB TIF)

Figure S7 **Primary BIAcore sensorgrams used to calculate equilibrium rate constants in** **Figure S3** **and** ***K*** **_d_ values in** **Figure 6** **.** Data analyses were undertaken as described in Materials and Methods. Similar results were obtained in two separate experiments carried out in duplicate. Kinetic fits in (A, C, and E) correlate well with equilibrium fits in (B, D, and F), respectively, as is expected for specific binding that follows the law of mass action.
(1.08 MB TIF)

Table S1 **STRADα interaction rate constants for MO25α (WT) and MO25α mutants.** *k*_a_ and *k*_d_ values were calculated from BIAcore sensorgrams in Figure S7. Error values are given in parentheses.
(0.05 MB PDF)

Text S1 **Purification and kinase activity assays of STRADα.**
(0.03 MB DOC)

## Abbreviations

PMSE: polyhydramnios, megalencephaly, symptomatic epilepsy
RMSD: root mean square deviation
SPR: surface plasmon resonance
