# Supplementary figures and images for: ATP and MO25α Regulate the Conformational State of the STRADα Pseudokinase and Activation of the LKB1 Tumour Suppressor
Source: PLoS Biol. 2009 Jun 9;7(6):e1000126. doi: 10.1371/journal.pbio.1000126 (PMC2686265; doi:10.1371/journal.pbio.1000126)

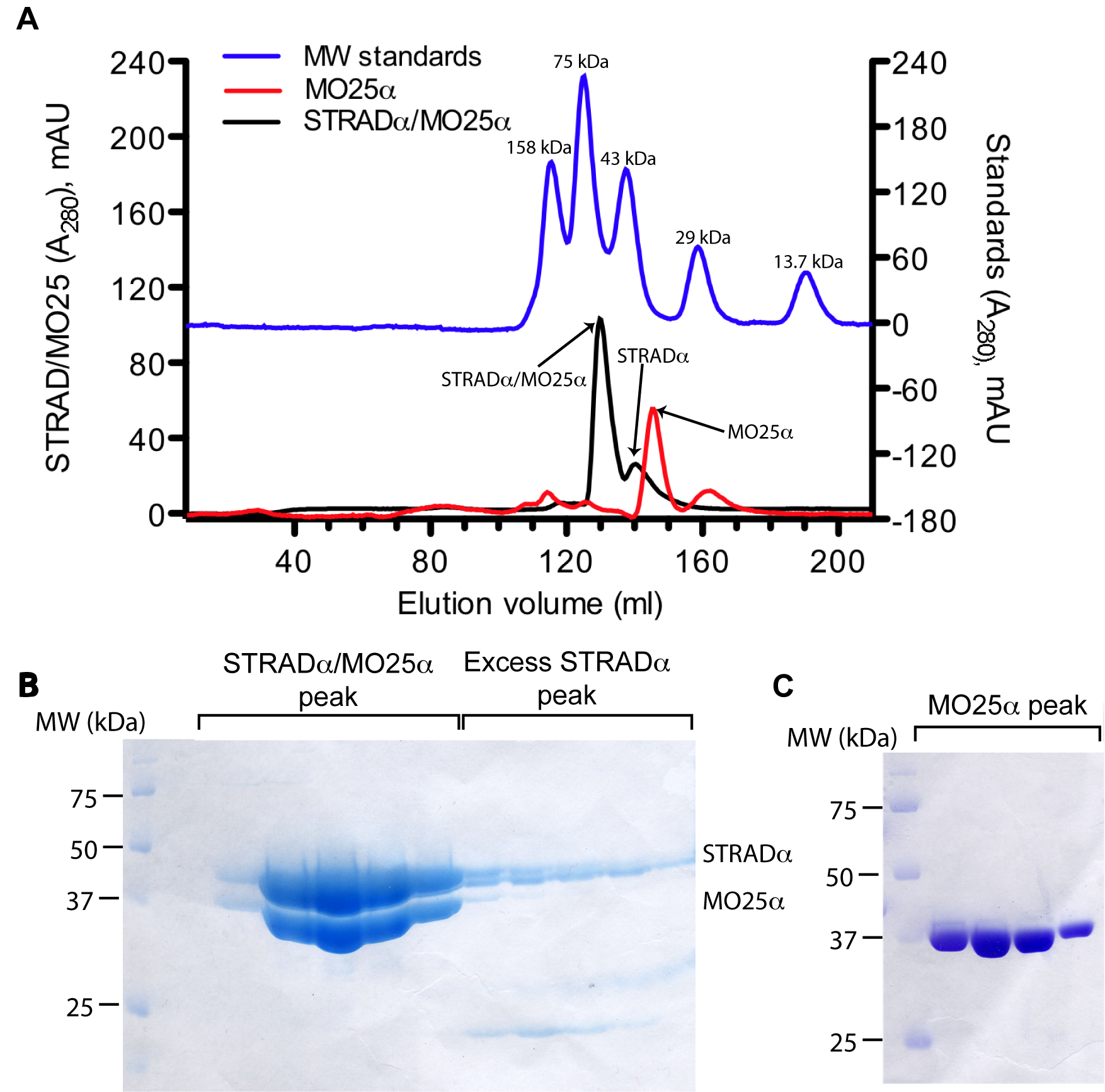

Supplement: Figure S1 — Isolation of the heterodimeric STRADα/MO25α complex. (A) Gel filtration profiles of His-STRADα/MO25α coexpressed in E. coli and crystallised in this study. The elution profile of separately expressed MO25α monomer as well as the molecular mass standards aldolase (158 kDa), conalbumin (75 kDa), ovalbumin (43 kDa), carbonic anhydrase (29 kDa), and ribonuclease A (13.7 kDa) are also shown. (B) We analysed, by SDS-PAGE, the fractions in which STRADα/MO25α dimer and MO25α monomer were eluted and stained with Coomassie Blue. There is no evidence for large molecular weight aggregates of His-STRADα/MO25α. In the His-STRADα/MO25α purification, a minor low molecular weight eluting shoulder to the main peak was found to consist of mainly uncomplexed His-STRADα. Because His-STRADα was the subunit used for nickel affinity purification of the complex, it will be expected to be present in excess. (1.99 MB TIF) [file pbio.1000126.s001.tif]

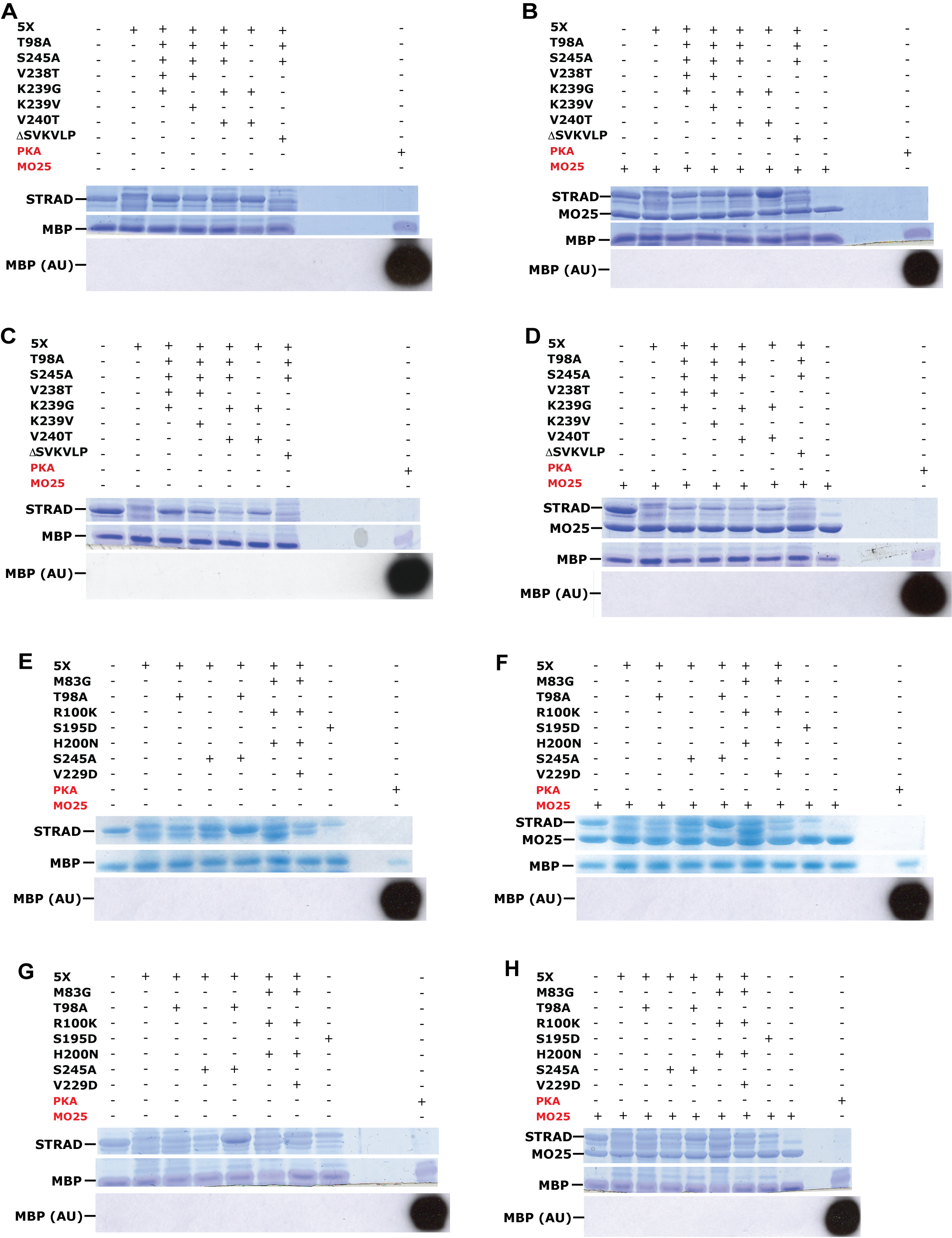

Supplement: Figure S2 — Attempts at reactivating the STRADα pseudokinase. The indicated STRADα (residues 59–431) active site mutants were expressed in E. coli and tested for kinase activity in the presence of 0.2 mM γ-32P-ATP and 10 mM magnesium acetate, (A) alone or (B) in the presence of MO25α. Similarly, in (C) and (D), the same mutations were tested in the absence of magnesium acetate. (E–H) STRADα active site mutants were combined with mutations/deletions from the P+1 site of the kinase. (E and F) were tested in the presence of magnesium acetate, whereas (G and H) were tested in the absence of Mg2+. In all cases, PKA assayed in the presence of Mg2+ was included as a positive control. (5X = T98A+R100K+G213D+L214F+R215G) (3.73 MB TIF) [file pbio.1000126.s002.tif]

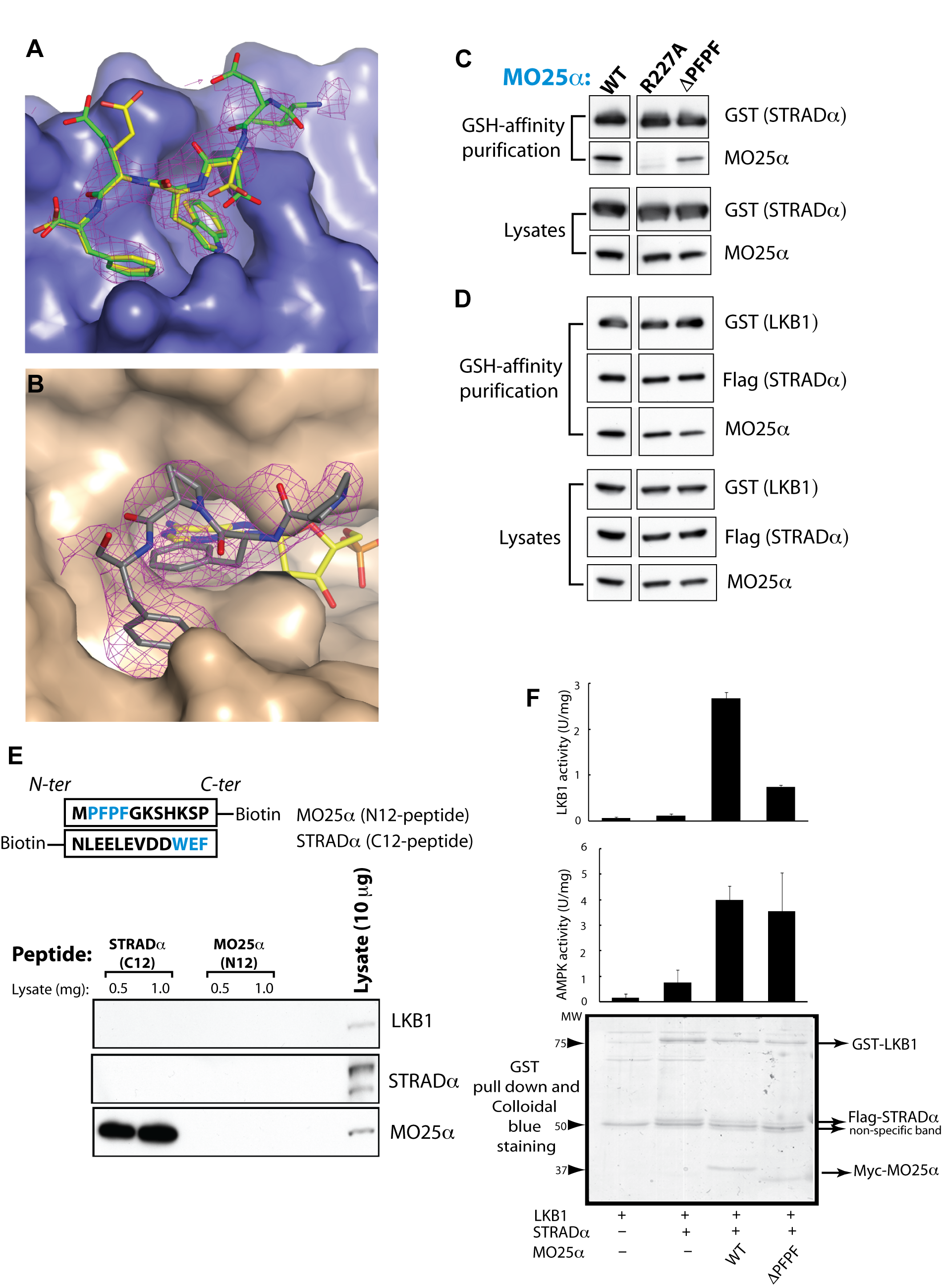

Supplement: Figure S3 — Characterisation of the MO25α PFPF motif, the STRADα WEF motif, and effects on LKB1 binding. (A) Comparison of STRADα WEF motif, binding to the MO25α WEF pocket. WEF motifs from the STRADα/MO25α complex structure and MO25α/peptide complex determined previously by Milburn et al. [16], are superimposed (RMSD = 0.3 Å over 35 atoms) and shown as stick models with green and yellow carbon atoms, respectively. Electron density maps (F o-F c are shown for the WEF motif determined in this study and contoured at 2.5σ). (B) The PFPF motif of MO25α binds to a STRADα hydrophobic pocket, near the ATP binding site. Electron density maps are displayed as described above. (C) The indicated constructs of GST-STRADα and untagged MO25α were expressed in 293 cells. Cells were lysed 36 h posttransfection and GST-STRADα was affinity purified on glutathione-Sepharose. The purified GST-STRADα preparation (upper panels), as well as the cell extracts (lower panel), was immunoblotted with the indicated antibodies. STRADα R227A mutant, unable to bind MO25α, was used as a control. (D) Wild-type GST-LKB1 and indicated forms of Flag-STRADα and untagged MO25α were cotransfected in 293 cells. Cells 36 h posttransfection were lysed, and GST-LKB1 was affinity purified on glutathione-Sepharose. The purified GST-LKB1 preparations (upper panels), as well as the cell extracts (lower panel), were immunoblotted with the indicated antibodies. (E) Either 0.5 or 1.0 mg of the indicated cell lysates were incubated with 5 µg of the indicated biotinylated peptides conjugated to Streptavidin-Sepharose. Following isolation and washing of the beads, the samples were subjected to SDS-polyacrylamide gel electrophoresis and immunoblotted with the indicated antibodies. (F) Activation of the bacterially expressed AMPK complex using wild-type or mutant LKB1/STRADα/MO25α complex. The purity of LKB1 complexes was analyzed by SDS-PAGE and colloidal blue staining. (3.82 MB TIF) [file pbio.1000126.s003.tif]

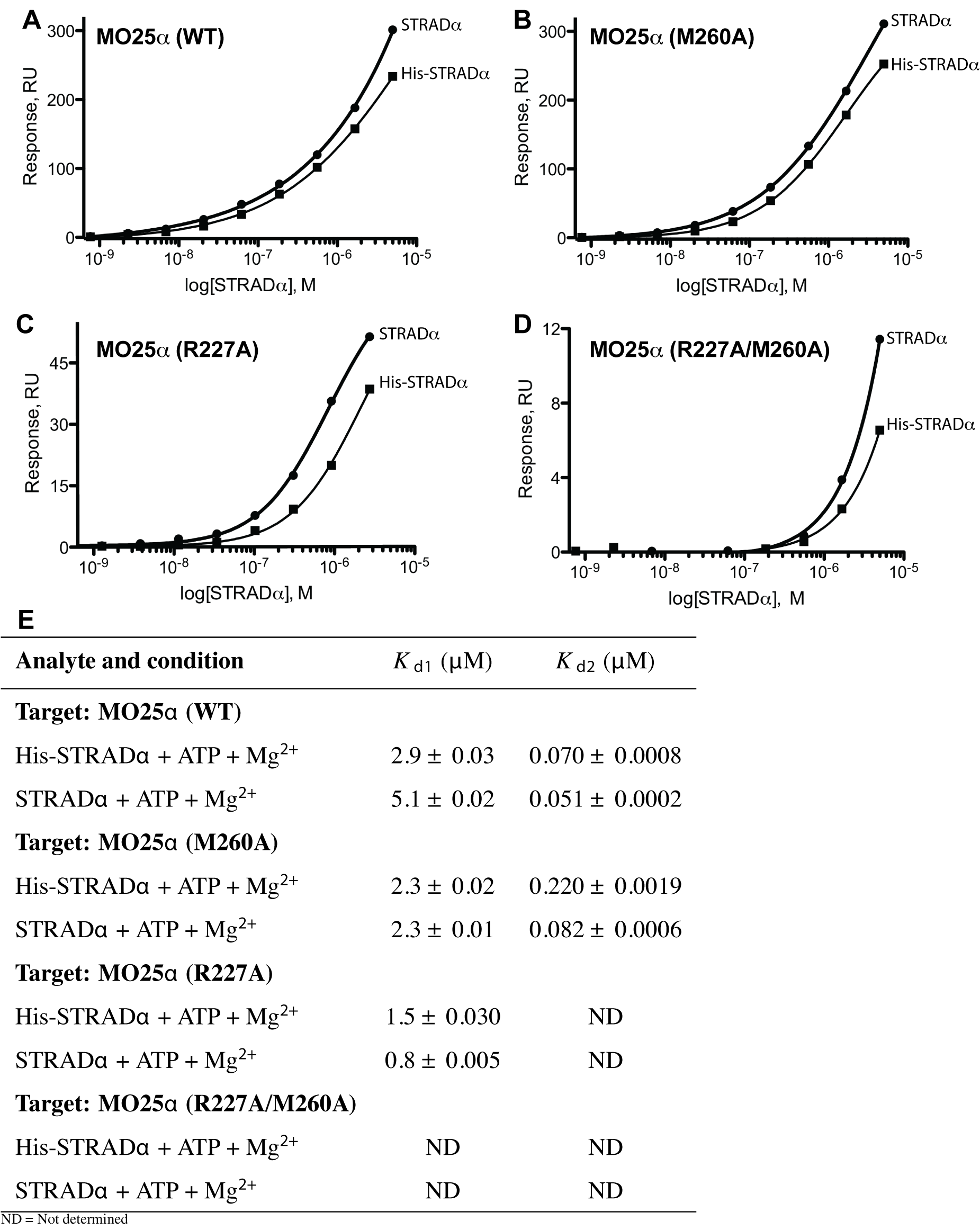

Supplement: Figure S4 — His-tagged STRADα and untagged STRADα bind MO25α with similar affinity. His-STRADα was treated in the presence or absence of His-TEV protease to remove the 6-His purification tag and then repurified using nickel agarose to remove His-TEV and any uncleaved His-STRADα (see Materials and Methods). Binding was assessed by SPR analyses where (A) MO25α(WT) (wild type), (B) MO25α(M260A), (C) MO25α(R227A), and (D) MO25α(R227A/M260A) were immobilised to a CM5 sensor chip. Equivalent concentrations of His-STRADα or untagged STRADα, were allowed to bind over 50 s by injecting different concentrations over a range of 0.4 nM to 5 µM, in the presence of 0.1 mM ATP and 1 mM MgCl2. Response level for specific binding of STRADα to MO25α was plotted against STRADα concentration (log scale), using a variable slope model (where appropriate) to determine the Hill slope from the data. (E). Reported K d values were calculated by measuring association (k a) and dissociation (k d) rates from the BIAcore sensorgram data shown in Figure S7 and Table S1, using Scrubber-2 software. K d values reported here were calculated as K d = k d/k a (see Materials and Methods). Equilibrium binding constants were also calculated from a saturation binding model, and similar values were obtained. (see Figure S7 and Materials and Methods). (0.66 MB TIF) [file pbio.1000126.s004.tif]

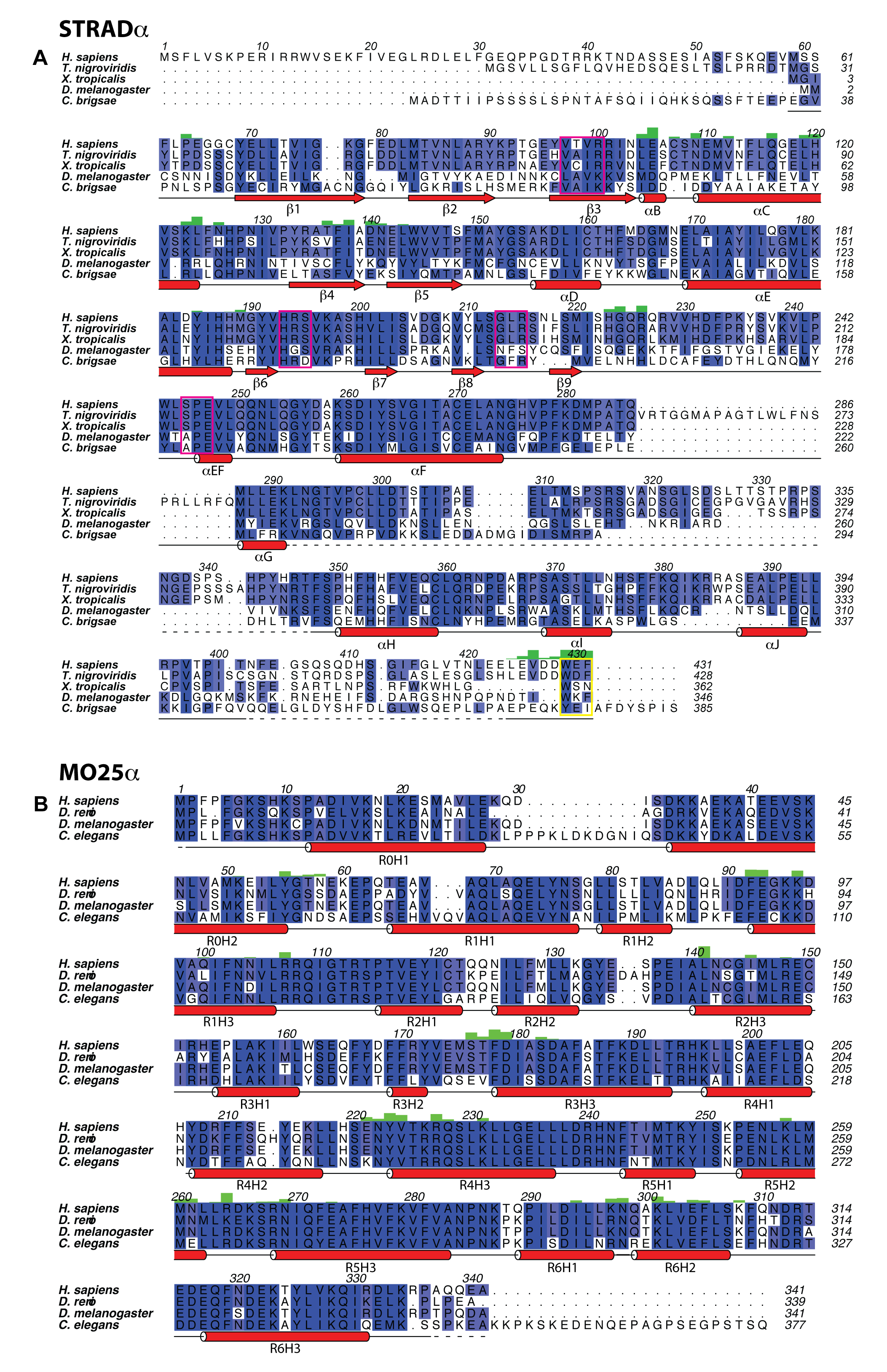

Supplement: Figure S5 — Sequence conservation of STRADα and MO25α. Sequence alignment (dark blue = conserved, white = not conserved) of STRADα (A) and MO25α (B) of the indicated species. Alignments were performed with MUSCLE and edited and displayed using ALINE (Charlie Bond and Alex Schüttelkopf). A graph of residues involved in STRADα/MO25α interaction against their contact area (green bars), is displayed. Height of the bar represents the contact area (atom pairs closer than 3.9 Å, analysed by CONTACT from the CCP4 package), divided by the molecular weight of the participating amino acid. Key STRADα catalytic motifs and the WEF motif are boxed. The secondary structure (analysed by DSSP) is shown in red. Dotted lines represent residues missing in our structural model. (1.93 MB TIF) [file pbio.1000126.s005.tif]

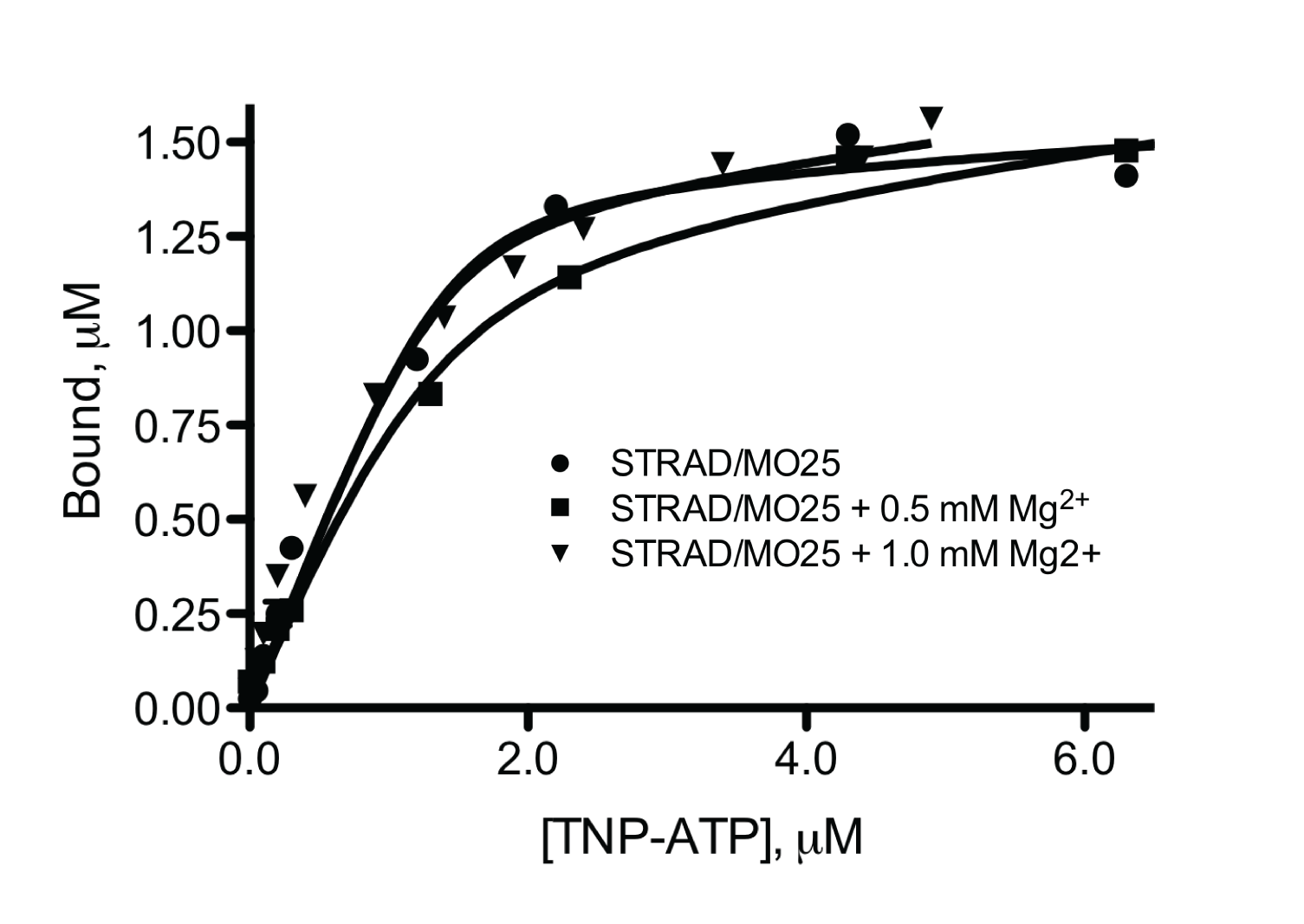

Supplement: Figure S6 — Binding of STRADα/MO25α complex to the ATP fluorescent analog TNP-ATP±MgCl2. Saturation binding experiments for STRADα/MO25α complex to TNP-ATP in the presence/absence of 0.5 mM and 1 mM MgCl2. Bound was defined as (F x/F max)[R], where F max and F x are maximal and fractional fluorescence (recorded at 540 nm), respectively, and [R] equals the binding capacity, defined by the enzyme concentration, fixed at 1.5 µM. Equilibrium binding curves were then fitted to the quadratic equation suitable for tight binding interactions with ligand depletion (see Materials and Methods). K d values were calculated as: 0.09±0.03 µM, 0.23±0.06 µM, and 0.09±0.04 µM for TNP-ATP, TNP-ATP+0.5 mM MgCl2, and TNP-ATP+1.0 mM MgCl2, respectively. Data shown are the average of two independent experiments. (0.30 MB TIF) [file pbio.1000126.s006.tif]

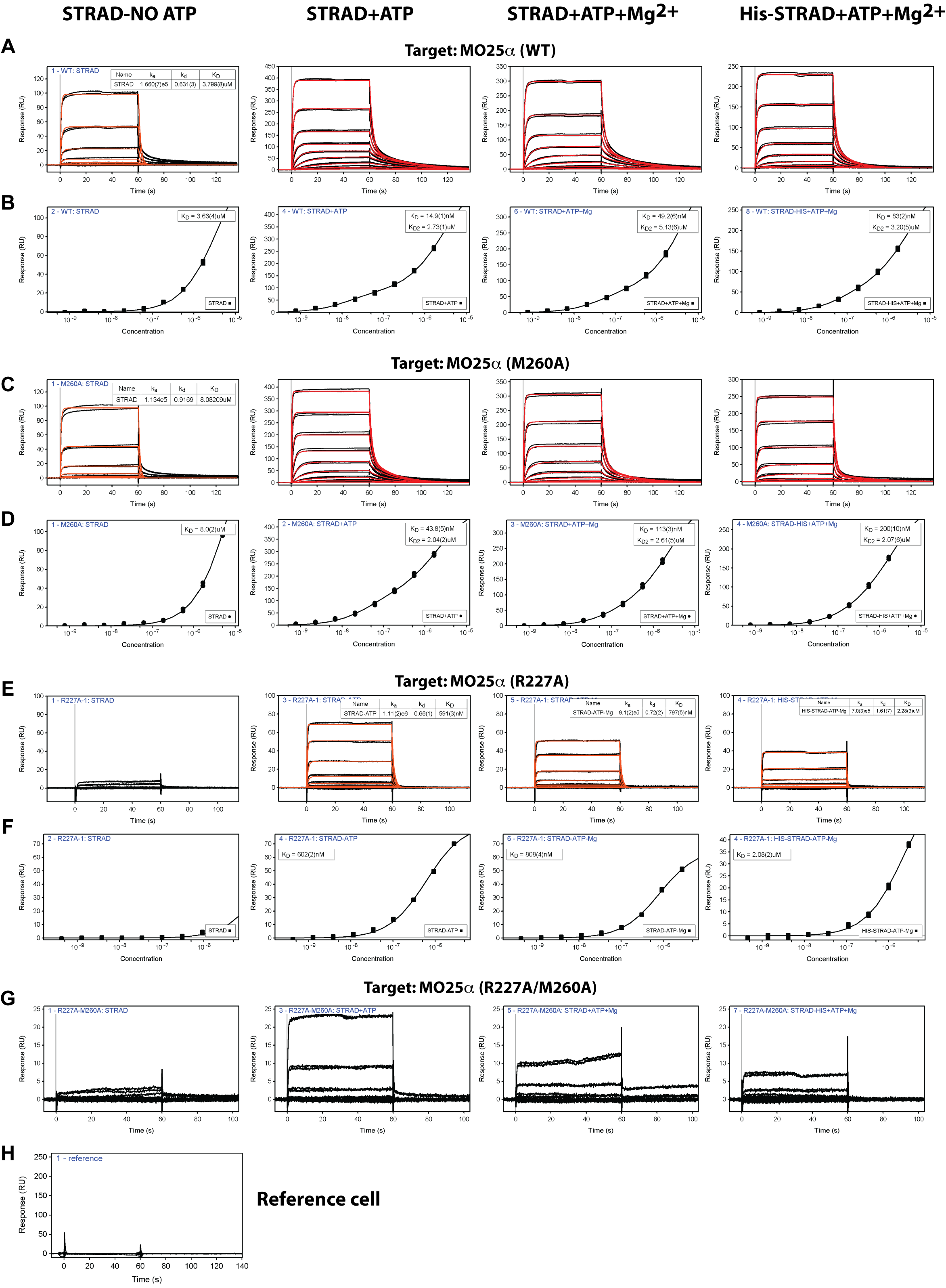

Supplement: Figure S7 — Primary BIAcore sensorgrams used to calculate equilibrium rate constants in Figure S3 and K d values in Figure 6 . Data analyses were undertaken as described in Materials and Methods. Similar results were obtained in two separate experiments carried out in duplicate. Kinetic fits in (A, C, and E) correlate well with equilibrium fits in (B, D, and F), respectively, as is expected for specific binding that follows the law of mass action. (1.08 MB TIF) [file pbio.1000126.s007.tif]
