# Supplementary material for: ATP and MO25α Regulate the Conformational State of the STRADα Pseudokinase and Activation of the LKB1 Tumour Suppressor
Source: PLoS Biol. 2009 Jun 9;7(6):e1000126. doi: 10.1371/journal.pbio.1000126 (PMC2686265; doi:10.1371/journal.pbio.1000126)
Supplement: Table S1 — STRADα interaction rate constants for MO25α (WT) and MO25α mutants. k a and k d values were calculated from BIAcore sensorgrams in Figure S7. Error values are given in parentheses. (0.05 MB PDF) [file pbio.1000126.s008.pdf]

Supplementary Table I

STRADα interaction rate constants for MO25α (WT) and MO25α mutants. k_a and k_d values were calculated from BIAcore sensograms in Supplementary Fig. 7. Error values are given in brackets.

**Analyte and condition**	k_{a1} ($M^{-1}s^{-1}$)	k_{d1} (s^{-1})	k_{a2} ($M^{-1}s^{-1}$)	k_{d2} (s^{-1})
**Target: MO25α (WT)**				
STRADα	1.7×10^5 (7.0×10^2)	0.63 (3.0×10^{-3})	ND	ND
STRADα + ATP	1.6×10^5 (5.8×10^2)	0.40 (1.3×10^{-3})	2.5×10^6 (1.1×10^4)	0.03 (0.8×10^{-4})
STRADα + ATP + Mg^{2+}	8.3×10^4 (3.5×10^2)	0.43 (1.4×10^{-3})	7.9×10^5 (2.9×10^3)	0.04 (8.8×10^{-5})
His-STRADα + ATP + Mg^{2+}	1.9×10^5 (1.4×10^3)	0.55 (3.8×10^{-3})	1.1×10^6 (9.9×10^3)	0.08 (4.8×10^{-4})
**Target: MO25α (M260A)**				
STRADα	1.1×10^5 (4.0×10^2)	0.92 (3.0×10^{-3})	ND	ND
STRADα + ATP	3.5×10^5 (1.9×10^3)	0.52 (2.9×10^{-3})	2.0×10^6 (1.2×10^4)	0.06 (2.2×10^{-4})
STRADα + ATP + Mg^{2+}	1.9×10^5 (7.0×10^2)	0.43 (1.7×10^{-3})	6.1×10^5 (3.8×10^3)	0.05 (2.3×10^{-4})
His-STRADα + ATP + Mg^{2+}	3.3×10^5 (2.4×10^3)	0.77 (4.6×10^{-3})	5.0×10^5 (3.6×10^3)	0.11 (5.3×10^{-4})
**Target: MO25α (R227A)**				
STRADα	ND	ND	ND	ND
STRADα + ATP	1.1×10^6 (2.0×10^3)	0.66 (1.0×10^{-3})	ND	ND
STRADα + ATP + Mg^{2+}	9.1×10^5 (2.0×10^3)	0.72 (2.0×10^{-3})	ND	ND
His-STRADα + ATP + Mg^{2+}	7.0×10^5 (3.0×10^3)	1.61 (7.0×10^{-3})	ND	ND
**Target: MO25α (R227A/M260A)**				
STRADα	ND	ND	ND	ND
STRADα + ATP	ND	ND	ND	ND
STRADα + ATP + Mg^{2+}	ND	ND	ND	ND
His-STRADα + ATP + Mg^{2+}	ND	ND	ND	ND

ND = Not determined
