# Supplementary material for: ATP and MO25α Regulate the Conformational State of the STRADα Pseudokinase and Activation of the LKB1 Tumour Suppressor
Source: PLoS Biol. 2009 Jun 9;7(6):e1000126. doi: 10.1371/journal.pbio.1000126 (PMC2686265; doi:10.1371/journal.pbio.1000126)
Supplement: Text S1 — Purification and kinase activity assays of STRADα. (0.03 MB DOC) [file pbio.1000126.s009.doc]

# SUPPLEMENTARY MATERIAL TO: ATP and MO25 regulate the conformational state of the STRADα pseudokinase and its activation of the LKB1 tumour suppressor.

### Elton Zeqiraj^1,2^, Beatrice Maria Filippi^2^, Simon Goldie^1,2^, Iva Navratilova^3^, Jerome Boudeau^2^, Maria Deak^2^, Dario Alessi^2^ and Daan M. F. van Aalten^1*^

^1^ Division of Molecular Microbiology,
College of Life Sciences, University of Dundee, Dundee DD1 5EH, Scotland.
^2^ MRC Protein Phosphorylation Unit,
College of Life Sciences, University of Dundee, Dundee DD1 5EH, Scotland.
^3^ Division of Biological Chemistry and Drug Discovery,
College of Life Sciences, University of Dundee, Dundee DD1 5EH, Scotland.
^*^ To whom correspondence should be addressed.
E-mail: dmfvanaalten@dundee.ac.uk, Fax: ++ 44 1382 385764

## Supplementary methods

#### Purification and kinase activity assays of STRADα

STRADα residues 59-431 preceded by a 6-His purification tag, were expressed in *E. coli* BL21(DE3)pLysS cells. The expression and purification procedure was followed as described in the Material and Methods section for STRADα/MO25α complex. Approximately 3-5 μg of PKA, wild-type or the indicated mutant His-STRADα were assayed in a 25 μl reaction mixture containing 50 mM Tris-HCl (pH 7.5), 0.1% (v/v) 2-mercaptoethanol, 0.1 mM EGTA, 10 mM magnesium acetate, and 0.2 mM [γ-^3^2P]ATP (5000 cpm/pmol) in the presence of 5 μg of bovine MBP. In assays where 3 μg of bacterially expressed MO25α was present, this was incubated with STRADα for 30 minutes at 4 ^o^C before the reaction was started. In assays where magnesium acetate was omitted, 2 mM EDTA was added instead. After incubation for 60 min at 30 ^o^C, reactions were terminated by the addition of 6 μl of LDS sample buffer (5 times concentrated) and reaction mixtures (30 μl) were electrophoresed on SDS-polyacrylamide gels. These were dried and analyzed by autoradiography to quantify STRADα autophosphorylation or MBP phosphorylation.
